# The 3D Brain Unit Network Model to Study Spatial Brain Drug Exposure under Healthy and Pathological Conditions

**DOI:** 10.1007/s11095-020-2760-y

**Published:** 2020-07-09

**Authors:** Esmée Vendel, Vivi Rottschäfer, Elizabeth C.M. de Lange

**Affiliations:** 1 Mathematical Institute, Niels Bohrweg 1, 2333CA, Leiden, The Netherlands; 2 Leiden Academic Center for Drug Research, Einsteinweg 55, 2333CC, Leiden, The Netherlands

**Keywords:** Brain extracellular fluid, pharmacokinetics, mathematical, model, drug binding, drug transport

## Abstract

**Purpose:**

We have developed a 3D brain unit network model to understand the spatial-temporal distribution of a drug within the brain under different (normal and disease) conditions. Our main aim is to study the impact of disease-induced changes in drug transport processes on spatial drug distribution within the brain extracellular fluid (ECF).

**Methods:**

The 3D brain unit network consists of multiple connected single 3D brain units in which the brain capillaries surround the brain ECF. The model includes the distribution of unbound drug within blood plasma, coupled with the distribution of drug within brain ECF and incorporates brain capillaryblood flow, passive paracellular and transcellular BBB transport, active BBB transport, brain ECF diffusion, brain ECF bulk flow, and specific and nonspecific brain tissue binding. All of these processes may change under disease conditions.

**Results:**

We show that the simulated disease-induced changes in brain tissue characteristics significantly affect drug concentrations within the brain ECF.

**Conclusions:**

We demonstrate that the 3D brain unit network model is an excellent tool to gain understanding in the interdependencies of the factors governing spatial-temporal drug concentrations within the brain ECF. Additionally, the model helps in predicting the spatial-temporal brain ECF concentrations of existing drugs, under both normal and disease conditions.

**Electronic supplementary material:**

The online version of this article (10.1007/s11095-020-2760-y) contains supplementary material, which is available to authorized users.

## Introduction

Insight into the spatial-temporal distribution of a drug within the brain is still limited, but very important for improved understanding of drug interaction with binding sites and ultimately drug effects and side effects. The blood-brain barrier (BBB) is a major barrier of the brain and separates the blood plasma in the brain capillaries from the brain extracellular fluid (brain ECF). The BBB has great impact on the relationship between drug concentration-time profiles (pharmacokinetics; PK) within the blood plasma and the brain ECF (see i.e. (1)). However, there is a lack of understanding of the mechanisms that may lead to local differences of brain ECF PK.

Drug distribution within the brain ECF is governed by many factors, including blood plasma PK in the brain capillaries, BBB transport, diffusion, brain ECF bulk flow as well as by specific and non-specific binding, as reviewed in (2). All of these factors may be locally different, for example by disease. First, brain capillary density may increase as a consequence of certain brain diseases, like Huntington’s disease (3,4), as the disease may induce new blood vessels to sprout, giving rise to a denser network of brain capillaries. On the other hand, brain capillary density may decrease by ageing (i.e (5,6)). Second, BBB transport may be affected under particular (disease) conditions. In many neurological diseases, disruption of the tight junctions leads to an increase in BBB transport of drugs that normally are impeded in their transport across the paracellular route (i.e. small hydrophilic drugs). In addition, expression and/or functionality of active (influx and efflux) transporters may be higher or lower, see (7) for a recent review on this topic. Third, brain ECF diffusion and bulk flow may be hindered by local disease: as a consequence of BBB disruption (by disease conditions), blood-derived cells and debris may leak into the brain ECF. The presence of these cells and debris within the brain ECF hinders diffusion within the brain ECF and interrupts the generation of brain ECF bulk flow (7). Finally, the density of specific and non-specific binding sites may differ per location within the brain (see e.g. (8) or Allen Brain Atlas for examples on concentrations of specific binding sites (receptors) at different locations within the (mouse) brain).

**Fig. 1:**
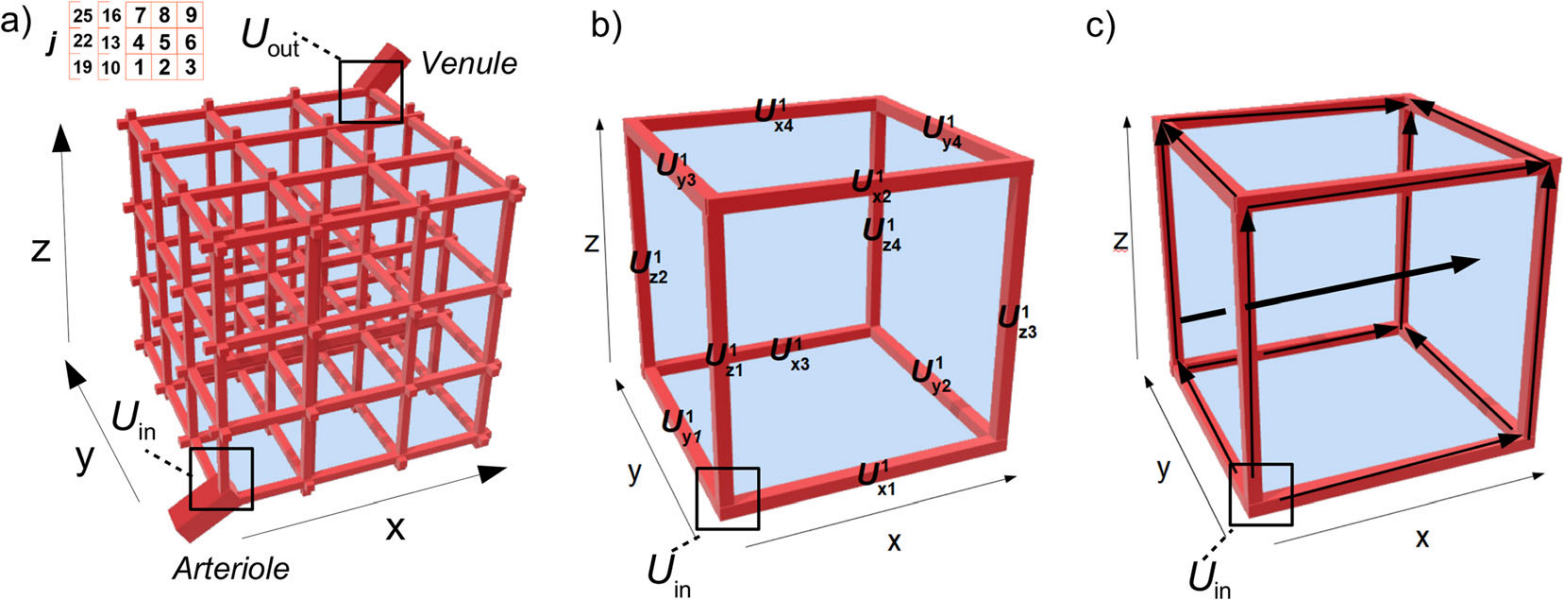
Sketch of the 3D model brain unit network. (**a**) The 3D brain unit network. The brain unit network consists of *N*3 single brain units. Here, *N* = 3. The single brain units are numbered j = 1-*N*^3^ (inset). In each brain unit, the brain capillaries surround the brain ECF. The brain capillaries (red) surround the brain ECF (blue) and denote the border of each unit. The brain capillaries are linked to an incoming arteriole and a draining venule. (**b**) The left front bottom 3D single brain unit is shown as an example as part of the 3D brain unit network. This unit consists of a blood-plasma-domain, which is contained in *U*_pl_ (red) and a brain-ECF-domain, contained in UECF (blue). The blood-plasma-domain is divided into several sub-domains: *U*_in_ is the domain where the dose of absorbed drug enters the 3D brain unit network, *U*_x1-x4_^j^, *U*_y1-y4_^j^ and *U*_z1-z4_^j^ are the domains representing the x-directed, ydirected and z-directed capillaries, respectively. Here, j = 1. c) Transport directions in the model. From Uin, drug is transported through the brain capillaries by the brain capillary blood flow in the direction indicated by the small arrows. Drug in the brain capillary blood plasma exchanges with the brain ECF by crossing the BBB. Drug within the brain ECF is, next to diffusion, transported by brain ECF bulk flow (indicated by the bold arrow).

In order to increase our understanding of drug distribution within the brain in health and disease conditions, we have developed a 3D network of single brain units that includes the brain capillary blood flow, passive (paracellular and transcellular) and active BBB transport, diffusion, brain ECF bulk flow and binding kinetics. The model builds on a single brain unit model that has recently been developed in 2D (9) and 3D (Vendel 2019, submitted to PLOS ONE). The 3D brain unit network consists of *multiple* connected 3D brain units, see Fig. 1 (left). This network is an improved representation of reality, because a) the brain capillaries are interconnected, and b) some brain capillaries are located more closely to the larger blood vessels (the arteriole and the venule) than others. Importantly, the network representation allows for the study of differences within the network, where one 3D brain unit may be assigned different properties (e.g. a higher specific binding site concentration) than another unit. Our model allows for the prediction of drug concentrations at any position within the 3D brain unit network, thereby providing insights into the spatial distribution of a drug within the brain. In this manuscript, we study the effects of brain capillary density, BBB transport, brain ECF diffusion and binding site density on drug distribution within the 3D brain unit network. We study the effect of local changes in these processes of brain drug distribution, as may occur in disease conditions or by differences in location within the brain, on drug distribution within the 3D brain unit network. To investigate how spatial drug distribution is affected by disease-induced changes in brain drug distribution processes, we compare drug distribution in a 3D brain unit network with `reference’ parameter values to drug distribution in a network with parameters that are different because of particular disease aspects. Below, in section 2, we first describe the 3D brain unit network and all the properties assigned to it. In section 3, we study drug distribution within the 3D brain unit network in health and disease conditions and in different locations within the brain. Finally, in section 4, we discuss and conclude our work.

## The 3D Brain Unit Network Model

We build a network of multiple connected single 3D brain units, based on the recent 3D brain unit model (submitted to PLOS ONE). The model describes drug distribution within a cubic domain that represents a piece of brain tissue. It includes the distribution of unbound drug within the blood plasma, coupled with the distribution of drug within the brain ECF and incorporates the brain capillar blood flow, passive paracellular and transcellular BBB transport, active BBB transport, drug diffusion and bulk flow within the brain ECF and the kinetics of drug binding to specific and non-specific binding sites. Here, we briefly summarize the 3D brain unit network model and, for full details, we refer to our earlier 3D brain unit model. The 3D brain unit network consists of multiple connected single 3D brain units. Each 3D brain unit is a cube, in which the brain capillaries surround the brain ECF. The brain capillaries within the network are linked to an incoming arteriole and a draining venule (Fig. 1a). From each brain capillary, drug is transported across the BBB into the brain ECF of all neighbouring 3D brain units. Drug within the brain ECF is transported by diffusion and bulk flow and freely exchanges between units. All assumptions made for the 3D brain unit network model are listed in Table 1.

**Table 1:** Model Assumptions

Brain capillaries	All brain capillaries are equal in size and area.
	The brain capillary blood flow velocity is constant in all brain capillaries.
	Diffusion is negligible compared to the blood flow.
	All drug is well mixed in the cross-capillary direction
	All drug is in unbound state.
Brain ECF	All drug within the brain distributes only within the brain ECF.
	The brain ECF bulk flow is unidirectional. In our model it points in the x-direction.
	Both specific and non-specific binding sites are exposed to brain ECF.
	Both specific and non-specific binding sites are evenly distributed over the 3D brain unit network without changing position.
	Drug binding is reversible.

### Model Formulation of the 3D Brain Unit Network

The 3D brain unit network is defined by a network of N^3^ brain units *U* = {(x,y,z) ∈ R^3^| 0 ≤ x ≤ Nx_r_ ∧ 0 ≤ y ≤ Ny_r_ ∧ 0 ≤ z ≤ Nz_r_}. The constants x_r_, y_r_ and z_r_ represent the length of one unit, which is defined as *d*_cap_ + 2*r*, with *d*_cap_ the brain intercapillary distance and *r* the brain capillary radius. The total length of the 3D brain unit network is given by N*d*_cap_ + 2N*r*. Capillary segments are defined for each 3D brain unit, see Fig. 1b. Each segment is named in the form *U*^j^_xi_, where j indicates unit number (see Fig. Figure 1a, inset) and xi indicates the capillary segment. For example, *U*^1^_x1_ describes capillary segment x1 in unit 1. In the current 3D brain unit network model, capillary segments of adjacent units are part of the same capillary. For instance, *U*^1^_y4_, *U*^2^_y3_,*U*^4^_y2_ and *U*^5^_y1_ belong to the same capillary.

Within the brain capillaries, diffusion is assumed to be negligible compared to the blood flow (Table 1). Therefore, within each capillary, drug is only transported in the direction of the flow. The brain ECF is continuous and brain ECF drug exchange between units occurs by diffusion (in all directions) and brain ECF bulk flow (in the x-direction only). The domain *U* is divided into the subsets *U*_pl_ ⊂ *U*, *U*_BBB_ ⊂ *U* and *U*_ECF_ ⊂ *U*, representing the brain capillaries, the BBB and the brain ECF, respectively, such that *U*=*U*_pl_ ∪ *U*_BBB_ ∪ *U*_ECF_^.^ Within *U*_pl_ we define the concentration of (unbound) drug by *C*_pl_. Within *U*_ECF_, we define the brain ECF concentrations of unbound drug, drug bound to specific binding sites and drug bound to non-specific sites by *C*_ECF_, *B*_1_ and *B*_2_.

### Description of Drug Distribution in *U*_pl_

We define the concentration of (unbound) drug within *U*_in_ as:

1 $$ {C}_{\mathrm{pl}}=\frac{F{k}_{\mathrm{a}} Dose}{V_{\mathrm{d}}\left({k}_{\mathrm{a}}-{k}_{\mathrm{e}}\right)}\left({\mathrm{e}}^{-{k}_{\mathrm{e}}\mathrm{t}}-{\mathrm{e}}^{-{k}_{\mathrm{a}}\mathrm{t}}\right)\mathrm{for}\ {C}_{\mathrm{pl}}\in {U}_{\mathrm{in}} $$

where F is the drug bio-availability, *k*_a_ the drug absorption rate constant, *k*_e_ the drug elimination rate constant, *Dose* the molar amount of orally administered drug, and *V*_d_ the drug distribution volume. This definition includes parameters related to oral administration. In case of single intravenous administration, all drug directly enters the blood.

Blood carrying the drug enters the 3D brain unit network in *U*_in_ and flows from there in the x-direction, y-direction and z-direction towards *U*_out_ (see Fig. 1c). We define:

2 $$ \frac{\partial {C}_{\mathrm{pl}}}{d\mathrm{t}}=-{v}_{\mathrm{blood}}\frac{\partial {C}_{\mathrm{pl}}}{\mathrm{\partial x}}\mathrm{for}\ {C}_{\mathrm{pl}}\in {U}_{xi}^j,\mathrm{for}\ i=1,..,4\ \mathrm{and}\ j=1,..,{N}^3 $$

3 $$ \frac{\partial {C}_{\mathrm{pl}}}{d\mathrm{t}}=-{v}_{\mathrm{blood}}\frac{\partial {C}_{\mathrm{pl}}}{\mathrm{\partial y}}\mathrm{for}\ {C}_{\mathrm{pl}}\in {U}_{yi}^j,\mathrm{for}\ i=1,..,4\ \mathrm{and}\ j=1,..,{N}^3. $$

4 $$ \frac{\partial {C}_{\mathrm{pl}}}{d\mathrm{t}}=-{v}_{\mathrm{blood}}\frac{\partial {C}_{\mathrm{pl}}}{\mathrm{\partial z}}\mathrm{for}\ {C}_{\mathrm{pl}}\in {U}_{zi}^j,\mathrm{for}\ i=1,..,4\ \mathrm{and}\ j=1,..,{N}^3 $$

where v_blood_ is the blood flow velocity within the brain capillaries and where the initial condition is given by

5 $$ {C}_{pl}\left(\mathrm{x},\mathrm{y},\mathrm{z},\mathrm{t}=0\right)=0 $$

### Description of Drug Distribution in *U*_ECF_

We describe the distribution of unbound and bound drug within *U*_ECF_ with the following system of equations:

6 $$ {\displaystyle \begin{array}{l}\frac{\partial {C}_{\mathrm{ECF}}}{\mathrm{\partial t}}={D}^{\ast }{\nabla}^2{C}_{\mathrm{ECF}}-{v}_{\mathrm{ECF}}\frac{\partial {C}_{\mathrm{ECF}}}{\partial x}-{k}_{1\mathrm{on}}{C}_{\mathrm{ECF}}\left({B}_1^{\mathrm{max}}-{B}_1\right)+{k}_{1\mathrm{off}}{B}_1\\ {}-{k}_{2\mathrm{on}}{C}_{\mathrm{ECF}}\left({B}_2^{\mathrm{max}}-{B}_2\right)+{k}_{2\mathrm{off}}{B}_2\\ {}\frac{\partial {B}_1}{\mathrm{\partial t}}={k}_{1\mathrm{on}}{C}_{\mathrm{ECF}}\left({B}_1^{\mathrm{max}}-{B}_1\right)-{k}_{1\mathrm{off}}{B}_1\\ {}\frac{\partial {B}_2}{\mathrm{\partial t}}={k}_{2\mathrm{on}}{C}_{\mathrm{ECF}}\left({B}_2^{\mathrm{max}}-{B}_2\right)-{k}_{2\mathrm{off}}{B}_2\end{array}} $$

with initial conditions

7 $$ {C}_{ECF}\left(\mathrm{x},\mathrm{y},\mathrm{z},\mathrm{t}=0\right)=0, $$

8 $$ {B}_i\left(\mathrm{x},\mathrm{y},\mathrm{z},\mathrm{t}=0\right)=0,i=1,2, $$

with D^*^ =$\frac{D}{\lambda^2}$, where *D* is the diffusion coefficient in a free medium and *λ* the tortuosity, *v*_ECF_ the (x-directed) brain ECF bulk flow velocity, *B*_1_^max^ the total concentration of specific binding sites, *k*_1on_ the association rate constant for specific binding, *k*_1off_ the dissociation rate constant for specific binding, ${B}_2^{\mathrm{max}}$ the total concentration of non-specific binding sites, *k*_2on_ the association rate constant for non-specific binding and *k*_2off_ the dissociation rate constant for non-specific binding.

### Boundary Conditions

We describe drug transport across the BBB as follows:

9 $$ \mathrm{f}\left(\mathrm{u},\mathrm{v}\right)=P\left(\mathrm{u}-\mathrm{v}\right)+\frac{T_{\mathrm{m}-\mathrm{in}}}{S{A}_{\mathrm{BBB}}\left({\mathrm{K}}_{\mathrm{m}-\mathrm{in}}+\mathrm{u}\right)}\mathrm{u}-\frac{T_{\mathrm{m}-\mathrm{out}}}{S{A}_{\mathrm{BBB}}\Big({\mathrm{K}}_{\mathrm{m}-\mathrm{out}}+\mathrm{v}}\mathrm{v} $$

with u = *C*_pl_,v = *C*_ECF_, *P* the BBB permeability, *T*_m-in_ the maximum rate of active influx, *T*_m-out_ the maximum rate of active efflux, *K*_m-in_ the concentration of drug at which half of *T*_m-in_ is reached, *K*_m-out_ the concentration of drug at which half of *T*_m-out_ is reached and *SA*_BBB_ a correction factor taking the BBB surface area into account.

Based on expression (9), BBB transport of unbound drug into *U*_ECF_ is described with (example for the x direction):

10 $$ {\displaystyle \begin{array}{l}-D\ast \frac{\partial {C}_{\mathrm{ECF}}}{\partial x}=\mathrm{f}\left({C}_{\mathrm{pl}},{\mathrm{C}}_{\mathrm{ECF}}\right),\mathrm{for}\ \left(\mathrm{x},\mathrm{y},\mathrm{z}\right)\in {U}_{\mathrm{BBB}},\mathrm{at}\ \mathrm{x}=\mathrm{r}+\mathrm{n}\left({\mathrm{x}}_{\mathrm{r}}+{2}_{\mathrm{r}}\right),\mathrm{for}\ \mathrm{n}=0,...,\mathrm{N}-1\\ {}D\ast \frac{\partial {C}_{\mathrm{ECF}}}{\partial x}=\mathrm{f}\left({\mathrm{C}}_{\mathrm{pl}},{\mathrm{C}}_{\mathrm{ECF}}\right),\mathrm{for}\ \left(\mathrm{x},\mathrm{y},\mathrm{z}\right)\in {U}_{\mathrm{BBB}}\ \mathrm{at}\ \mathrm{x}=\mathrm{r}+\mathrm{n}\left({\mathrm{x}}_{\mathrm{r}}+2r\right), for\ n=1,...,N.\end{array}} $$

For drug transport into *U*_pl_, we use the reverse of expression (10).

We describe drug concentrations at the sides of *U*_pl_ and U_ECF_ with no-flux boundary conditions. At the sides of *U*_pl_,we describe drug concentrations with (example for the x direction):

11 $$ \frac{\partial {C}_{\mathrm{pl}}}{\mathrm{\partial x}}=0 $$

for (x,y,z) ∈ *U*_pl_\*U*_out_∩*∂U*, for x = 0 and x = Nx_r_.

At the sides of *U*_ECF_, we describe drug concentrations with:

12 $$ n\cdot \nabla {C}_{\mathrm{ECF}=0\ \mathrm{for}\ \left(\mathrm{x},\mathrm{y},\mathrm{z}\right)\in {U}_{\mathrm{ECF}}\cap \partial U}\kern0.5em $$

where n is the normal vector on *U*_*ECF*_∩*∂U.*

### Model Parameter Values and Units

The 3D brain unit network model dimensions are, like for the previous brain unit model (9), based on the properties of the rat brain. Within the 3D brain unit network, blood plasma PK is described using eqs. (1)–(5) with boundary conditions described in eqs. (10)–(12), while brain ECF PK is described with eqs. (6)–(8) with boundary conditions described in (10) and (13).

For our model analysis, we use, unless otherwise indicated, parameter values that are in the middle of the physiological ranges given in Table 2 (see also (9)). The reference parameter values of the drug are given in Table 3. These values, as shown in Table 3, are used in all simulations, unless stated differently. The parameter values may depend on their (x,y,z)-position within the 3D brain unit network. For example, in section 3.3, B_1_^max^ varies per unit and is assigned different values depending on the position within the 3D brain unit network.

**Table 2:** The Reference 3D Brain unit Model Parameters and their Units, for Rat Brain

Parameter	Unit	Value range
*F*, bioavailability	–	0–1
*Dose*	μmol	10^−1^-5·10^3^
*V*, distribution volume	L	3–5·10^3^
*k*_a_, absorption rate constant	s^−1^	0–2·10^−3^
*k*_e_, elimination rate constant	s^−1^	10^−1^ - 5·10^−3^
*d*_cap_, intercapillary distance	m	2·10^−5^-7·10^−5^
*r*, brain capillary radius	m	0.8–4.8·10^−6^
v_blood_, brain capillary blood flow velocity	m s^−1^	0.5–50·10^−4^
${D}^{\ast }=\frac{D}{\lambda^2}$, effective diffusion coefficient	m^2^ s^−1^	10^−11^–10^−10^
v_ECF_, brain ECF bulk flow velocity	m s^−1^	5·10^−8^-5·10^−6^
*P*, 3D passive BBB permeability^a^	m s^−1^	10^−10^–10^−5^
*T*_m − in_, maximal active influx rate	μmol s^−1^	10^−8^–10^−5^
*K*_m − in_, concentration needed to reach half of *T*_m − in_	μmol L^−1^	10^1^–10^4^
*T*_m − out_, maximal active efflux rate	μmol s^−1^	10^−8^–10^−5^
*K*_m − out_, concentration needed to reach half of *T*_m − out_	μmol L^−1^	10^1^–10^4^
*SA*_BBB_, surface area of the BBB	m^2^	1.25·10^−10^
${B}_1^{\mathrm{max}}$,total concentration specific binding sites	μmol L^−1^	1·10^−3^-5·10^−1^
*k*_1on_, specific association constant	(μmol L^−1^s)^−1^	10^−4^-10^2^
*k*_1off_, specific dissociation constant	s^−1^	10^−6^-10^1^
${B}_2^{\mathrm{max}}$, total non-specific binding sites	μmol L^−1^	1·10^1^–5·10^3^
*k*_2on_, non-specific association constant	(μmol L^−1^s)^−1^	10^−6^-10^1^
*k*_2off_, non-specific dissociation constant	s^−1^	10^−4^-10^3^

The physiological range of values of the parameters is given. These are based on references from the literature, see (9) for references

^a^This value is the apparent (experimentally measured) overall passive permeability

**Table 3:** 3D Brain Unit Model Reference Parameters and their Units

Parameter	Unit	Value
*N*	–	3·10^0^
*F*	–	1·10^0^
Dose	μmol	50·10^0^
*k*_a_	s^−1^	2·10^−4^
*k*_e_	s^−1^	5·10^−5^
*V*	L	2·10^1^
*d*_cap_	m	50·10^−6^
*r*	m	2.5·10^−6^
*v*_blood_	m s^−1^	5·10^−4^
${D}^{\ast }=\frac{D}{\lambda }$	m^2^ s^−1^	0.5 10^−10^
*v*_ECF_	m ^s-1^	0.5·10^−6^
*P*	m s^−1^	1·10^−**8**^
*T*_m-in_	μmol s^−1^	1·10^−7^
*K*_m-in_	μmol L^−1^	1·10^2^
*T*_m-out_	μmol s^−1^	1·10^−7^
*K*_m-out_	μmol L^−1^	1·10^2^
*SA*_BBB_	m^2^	1·10^−10^
${B}_1^{\mathrm{max}}$	μmol L^−1^	5·10^−2^
*k*_1on_	(μmol L^−1^s)^−1^	1·10^0^
*k*_1off_	s^−1^	1·10^−2^
${B}_1^{\mathrm{max}}$	μmol L^−1^	5·10^1^
*k*_2on_	(μmol L^−1^s)^−1^	1·10^−2^
*k*_2off_	s^−1^	1·10^−0^

## Model Results

Prior to model analysis, the system of equations and boundary conditions are nondimensionalised by scaling all variables by the typical scales given in Table 3 (see Appendix 1 for details). Then, the nondimensionalised system is spatially discretised with a well-established numerical procedure using finite element approximations (10). The results are presented using the parameters with dimensions. The simulation output includes the concentrations of free, specifically bound and non-specifically bound drug, given in μmol L^−1^ overtime.

**Fig. 2:**
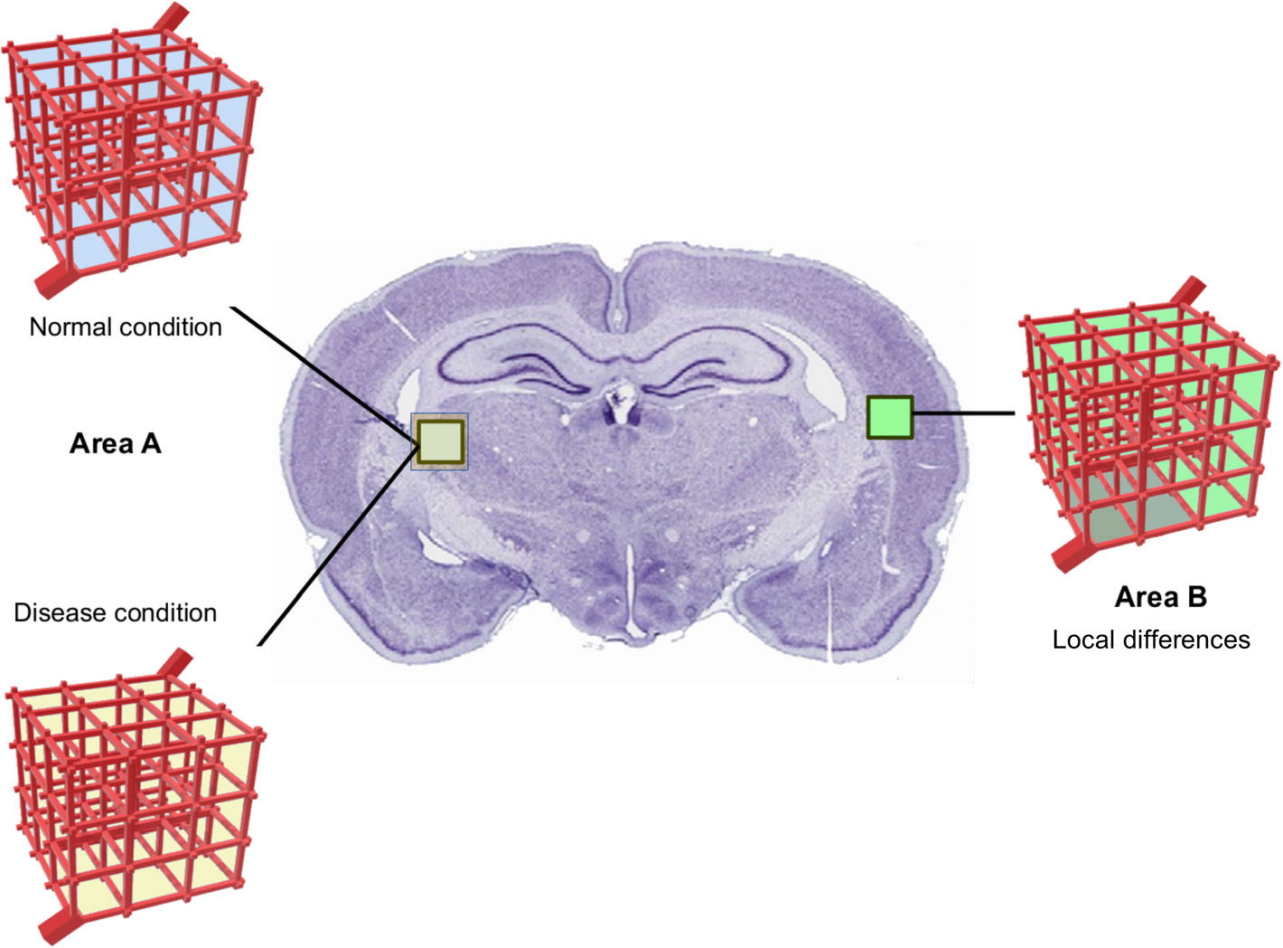
The 3D brain unit network that may represent different areas of the rat brain. The brain unit network with reference properties, with parameter values corresponding to the reference values given in Table 3, (left top) represents a normal condition. The properties of the 3D brain unit network may change as a consequence of local disease (left bottom) or by differences in location (right). Local differences in properties may also exist within the 3D brain unit network, as shown on the right. There, the dark green area indicates an area with different properties (i.e. higher concentration of specific binding sites) than the surrounding area network, i.e. specific binding sites may be concentrated within a particular area of the network, see Fig. 2 (right). We show the impact of brain capillary density (section 3.1), disruption of BBB transport (section 3.2) and differences in drug target concentrations (section 3.3) on local drug concentrations and drug distribution within the brain ECF (brain ECF PK). In section 3.4 we vary multiple properties and study their (combined) effect on drug concentrations within the brain ECF. In sections 3.2–3.4, we summarize the PK for each situation by the maximal attained concentration, *C*_max_, and *t*_max_ (time needed to attain *C*_max_) at various points in the network. We use *C*_max_,ECF, *C*_max_,B1, *t*_max_,ECF and *t*_max_,B1 for the *C*_max_ and *t*_max_ of *C*_ECF_ and *B*_1_, respectively. Distribution plots of the drug are given for cross-sections of the 3D brain unit network for various times.

In the following sections, we compare a 3D brain unit network with default properties, i.e. with parameter values corresponding to the reference values given in Table 3 (`normal condition’), to a 3D brain unit network with other properties, as may be induced by disease conditions (i.e. disruption of BBB transport) or location (i.e. a binding site density that differs per location), see Fig. 2. There, local differences may also exist *within* the 3D brain unit network, i.e. specific binding sites may be concentrated within a particular area of the network, see Fig. 2 (right). We show the impact of brain capillary density (section 3.1), disruption of BBB transport (section 3.2) and differences in drug target concentrations (section 3.3) on local drug concentrations and drug distribution within the brain ECF (brain ECF PK). In section 3.4 we vary multiple properties and study their (combined) effect on drug concentrations within the brain ECF. In sections 3.2–3.4, we summarize the PK for each situation by the maximal attained concentration, *C*_max_, and *t*_max_ (time needed to attain *C*_max_) at various points in the network. We use *C*_max_,_ECF_, *C*_max,B1_, *t*_max,ECF_ and *t*_max,B1_ for the *C*_max_ and *t*_max_ of *C*_ECF_ and *B*_1_, respectively. Distribution plots of the drug are given for cross-sections of the 3D brain unit network for various times.

### Simulated Changes in Brain Capillary Density

**Fig. 3:**
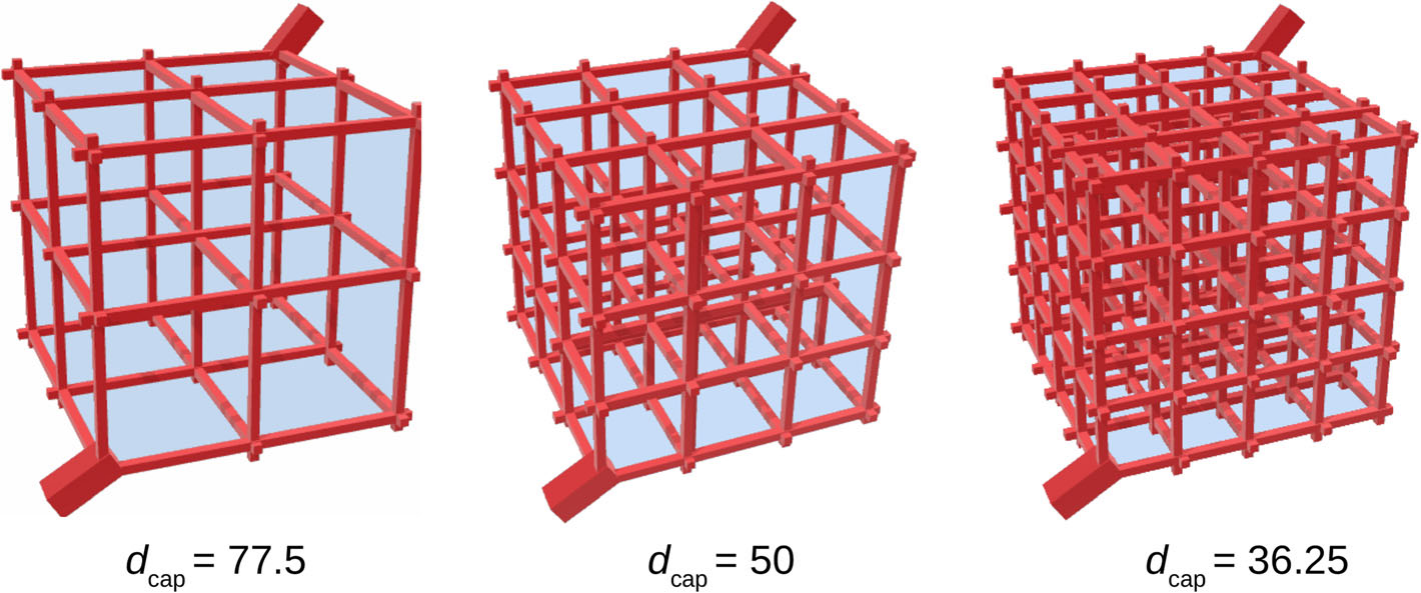
Geometries of 3D brain unit networks with varying capillary density. Left: decreased brain capillary density, middle: reference brain capillary density, right: increased brain capillary density. The distances between the capillaries, *d*_cap_ are set at 77.5, 50 and 36.25 μm.

**Fig. 4:**
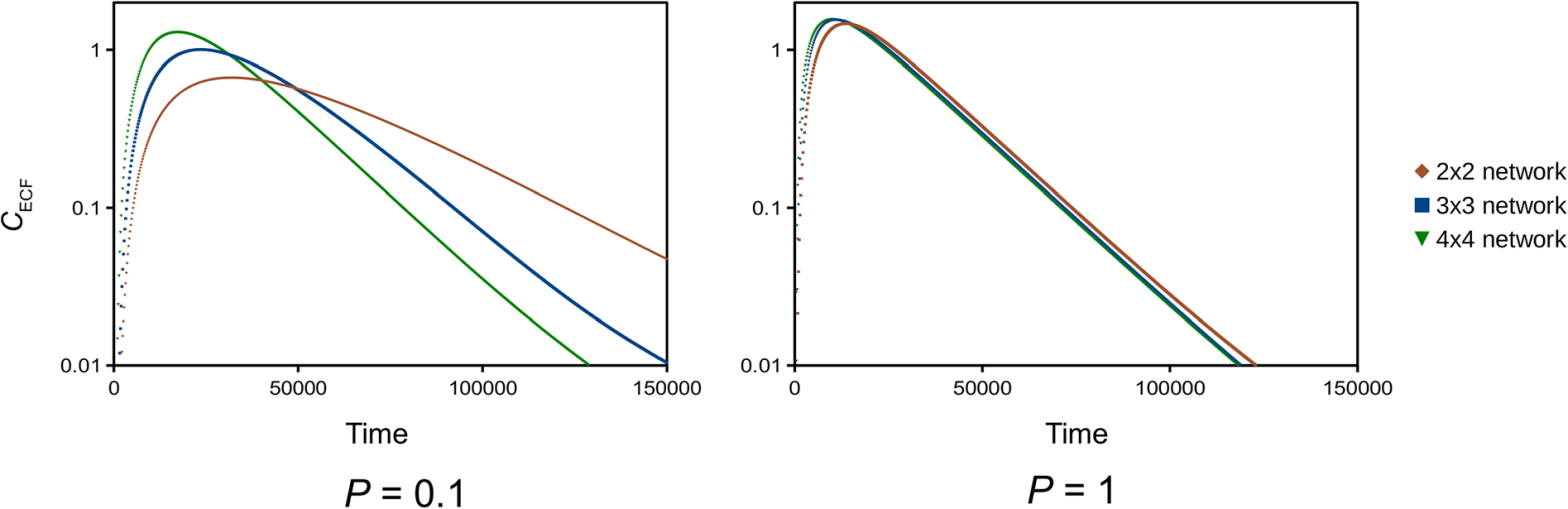
Effects of brain capillary density on the concentration of unbound drug within the brain ECF. The BBB permeability *P* is changed from 0.1·10^−7^ m s^−1^ to 1·10^−7^ m s^−1^, all other parameters are as in Table 3. *C*_ECF_ is measured at the middle of the unit bordering *U*_out_ in all configurations.

We evaluate the effect of brain capillary density on drug concentrations within the brain ECF. In Fig. 3, example geometries of 3D networks with different brain capillary densities are shown. There, brain capillary density is changed by varying *d*_cap_, while we leave the total size of the network unchanged. Figure 4 shows the effects of brain capillary density on *C*_ECF_ for different values of the passive BBB permeability, *P*. For proper comparison, *C*_ECF_ is measured on similar points for all brain capillary densities: in the middle of the right upper back unit, which is the unit next to the venule. When *P* is set at its reference value (*P* = 0.1 · 10^−7^ m s^−1^, as in Table 3), *C*_ECF_ increases with brain capillary density: with a higher brain capillary density, higher values of *C*_ECF_ are attained at earlier times. Moreover, *C*_ECF_ decreases more quickly when the brain capillary density is high than when it is low. On the other hand, when *P* is high (*P* = 1 · 10^−7^ m s^−1^), brain capillary density hardly affects *C*_ECF_ (Fig. 4, right): a decrease in brain capillary density leads to an only slightly lower value of *C*_max_,_ECF_ and an only slightly higher value of *t*_max_,_ECF_, while an *increase* in brain capillary density has no effect. This can be intuitively explained: with a high BBB permeability, drug quickly equilibrates between blood plasma and brain ECF as if it were one domain. In contrast, with a low permeability, exchange between blood plasma and brain ECF is limited and drug equilibration is slow. Then, the brain capillary density, and the increase in brain capillary surface, increases the extent of drug within the blood plasma that can be presented to the brain ECF.

### Simulated BBB Functionality in Health and Disease Conditions

Here, we study the effect of changes in parameters related to BBB transport on drug concentrations within the brain ECF. Table 4 summarizes how three types of BBB transport (passive (paracellular) transport, active influx and active efflux) are affected by changes in properties as induced by a few common brain diseases. Increases in passive (paracellular) BBB transport occur in all listed brain diseases. In addition, BBB active influx and efflux may increase or decrease under disease conditions. The areas of the brain that are affected differ per disease condition, as is summarized in Table 5 . It is important to note that the effect of disease-induced changes in BBB permeability on drug concentrations within the brain ECF also depends on the properties of the drug. An increase in passive (paracellular) BBB permeability mostly affects the transport of compounds that depend more on the paracellular route to get into and out of the brain. In addition, compounds that are not actively transported are unaffected by changes in active influx or active efflux.

**Table 4:** Changes in Properties of the BBB as Reported in Health and Under Specific Disease Conditions

Process	AD	ALS	Epilepsy	MS	Stroke	Tumour	PD
Passive transport							
(paracellular)	+	+	+	+	+	+	+
Active influx	+	?	?	?	?	+	?
Active efflux	±	+	+	?	–	+	+/

See i.e. (7,23) for some excellent reviews on this topic. It is shown how BBB trancellular transport, paracellular transport, active efflux and active efflux are affected in brain diseases compared to healthy conditions. There, no distinction is made between individual transporter types, but it is shown for active influx and active efflux in general. This is shown for Alzheimer’s Disease (AD) (see i.e. (11,12)). amyotrophic lateral sclerosis (ALS) (see i.e. (13)) epilepsy (15), multiple sclerosis (MS) (16), Parkinson’s Disease (PD) (17–19), stroke (20,21) and tumour (i.e. (22)). A + indicates an increase of the extent of the BBB transport process that is associated with the disease, while a - indicates a decrease. A ± indicates that both increases and decreases have been observed as a consequence of the disease. Finally, a? indicates that disease-induced changes on the BBB transport process are not known

**Table 5:** Areas of the Brain, where the BBB is affected per Disease Condition

Disease	Affected area
AD	Cortex and Hippocampus (11,12)
ALS	Medulla and Spinal Cord (13,14)
Epilepsy	Pariental gyrus and cortex (15)
MS	White matter (16)
PD	Midbrain (17), striatum (18), subthalamic nucleus (19)
Stroke	Site of stroke (20,21)
Tumour	Site of tumour (i.e. (22))

AD = Alzheimer’s disease, MS = multiple sclerosis, ALS = amyotrophic lateral sclerosis, PD = Parkinson’s Disease. Adapted from (7)

To gain information on the effect of disease-induced changes in BBB permeability on brain ECF PK for all types of drugs, we have studied the effect of all possible combinations of *P*, *T*_m-in_ and *T*_m-out_ on brain ECF PK within the 3D brain unit network model. There, brain ECF PK within the middle 3D brain unit is quantified by *C*_max_,_ECF_ and *t*_max,ECF_. A description of the main fundings of Table 6 is now given. An increase in *P* generally correlates with an increase in *C*_max_,_ECF_, except for when *T*_m-in_ ≥ *T*_m-out_ and with *T*_m-in_ > 0.1 − 10^−7^ μmol s^−1^, when an increase in *P* correlates with a decrease in *C*_max_,_ECF_. Acstive inux increases *C*_max,ECF_, but has less effect when the BBB is highly spermeable to the drug, as drug can easily diffuse across the BBB back into the blood plasma. In similar fashion, active efflux decreases *C*_max_,_ECF_, but less so in the presence of a high value of *P*. Interestingly, in the presence of identical active transport rates (*T*_m-in_ = *T*_m-out_ ≠0), *C*_max_,_ECF_ is *larger* compared to the reference’ state with no active transport (*T*_m-in_ = 0 and *T*_m-out_ = 0), except for when *T*_m-in_ = *T*_m-out_ = 0.1 − 10^−7^ mol s^−1^ and *P* = 1 · 10^−7^ m s^−1^. BBB transport parameters also affect *t*_max,ECF_. An increase in *P* or increase in *T*_m-out_ goes along with a smaller *t*_max_,_ECF_. In contrast, the value of *T*_m-in_ hardly affects *t*_max,ECF_.

**Table 6:**
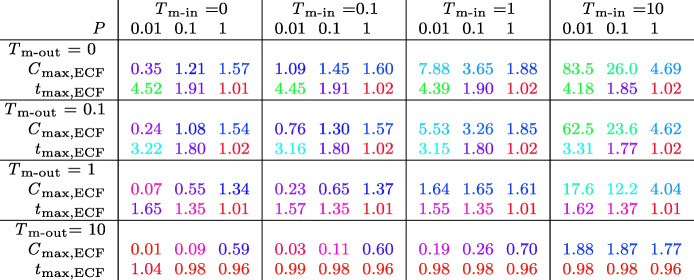
Impact of BBB Transport Parameters on Brain ECF PK of Unbound Drug

Here *C*_ECF_ is studied in the middle of the domain. The effects of *T*_m-in_(given in 10^−7^ μmol s^−1^), *T*_m-out_(given in 10^−7^ μmol s^−1^) and *P*(given in 10^−7^ m s^−1^) on *C*_max_,ECF (given in μmol L^−1^) and *t*_max_,ECF (given in 10^4^ s) are shown. Colours are added to increase the readability of the table. Red indicates the lowest value and green indicates the highest value. The values in between are coloured according to a 20-shades red-to-green colour bar based on the log values of the data

Next, we show the drug distribution within the 3D brain unit network for certain specific choices of parameters at t = 50. Figure 6 shows how changes in total BBB permeability and/or active influx affect *C*_ECF_. With a high value of *P* and/or with a high value of *T*_m-in_, values of *C*_ECF_ increase. In the presence of active influx, local differences in *C*_ECF_ are seen: concentrations are slightly higher in the upper back than in the front brain units in the presence of active influx. In addition, values of *C*_ECF_ are higher at locations close to the blood plasma. Interestingly, in the presence of a high value of *P*, a high value of *T*_m-in_ decreases *C*_ECF_ (brighter blue colours in Fig. 7, bottom right). Fig. 7 shows the effect of changes in total BBB transport combined with changes in active efflux on *C*_ECF_. The presence of active efflux decreases *C*_ECF_. In case of a low value of *P*, *C*_ECF_ is already low and the effect of changes in *T*_m-out_ on *C*_ECF_ is negligible. Interestingly, in the presence of a high value of *P* and a high value of *T*_m-out_ (Fig. 7, bottom right), values of *C*_ECF_ increase within each unit in the direction of the brain ECF bulk flow. In conclusion, we have shown that an increase in BBB active influx, as may happen in Alzheimer’s Disease, correlates with an increase in *C*_max,ECF_, while an increase in BBB active efflux, as may happen in amyotrophic lateral sclerosis and epilepsy, correlates with a decrease in both *C*_max_,_ECF_ and *t*_max_,_ECF_. If both active influx and active efflux are affected, like may be the case in brain tumours, the effects on both *C*_max_,_ECF_ and *t*_max_,_ECF_ depend on the rate of active influx and active efflux under healthy conditions and on the BBB permeability. Increases in BBB (paracellular) permeability, as occurs in all mentioned brain diseases (Table 4) but has most impact on drugs that have difficulties crossing the BBB, increases *C*_max_,_ECF_ and decreases *t*_max_,_ECF_.

This also means that drugs that easily cross the BBB are less impacted by disease-induced changes in BBB permeability.

### Simulated Changes in Specific Binding Site Density

Next, we study the effect of spatial differences in specific binding site (receptor) concentrations on brain ECF PK within the 3D brain unit network, which may represent different areas of the brain. Table 7 shows how concentration levels of various receptors differ over several brain areas. For example, dopamine receptor D2 (D2R) concentrations are generally highest in the striatum, while in the hippocampus, dopamine receptor concentrations are negligible.

**Table 7:** Spatial Differences in Brain Binding Site Concentrations. GR = Glucocorticoid receptor, MR = Mineralocorticoid receptor, D1R = Dopamine receptor D1, D2R = Dopamine receptor D2, 5-HT3AR = serotonine receptor type 3, CB1 = cannabinoid receptor type 1. Signs are based on raw expression values given by Allen Brain Atlas, unless indicated otherwise. – –= < 0.1, − = 0.1–0.5, ±  = 0.5–1.5, + = 1.5–5,++ = 5–10,+++= > 10. All values are based on binding site concentrations within the mouse brain. Data for 5-HT3AR are taken directly from (24), where ++, ± and - symbols refer to the signal intensities of 5-HT3AR linked to green fluorescent protein (GFP) in the corresponding regions of the brain

Receptors	Cortex	Hippocampus	Pons	Cerebellum	Striatum
CB1	++	++	+	+++	+++
D1R	±	±	–	±	+++
D2R	±	–	±	±	++
GR	±	±	–	±	–
MR	–	±	–	–	–
5-HT3AR	++	++	±	–	+±

To gain insight into the effect of (specific) binding site concentration on brain ECF PK for all types of drug, we first study the effect of all possible combinations of *B*_1_^max^, *k*_1on_ and *k*_1off_ on brain ECF PK within the 3D brain unit network. *Within* the 3D brain unit network, we keep all parameters constant. Tables 8 and 9 summarize the PK for each situation by *C*_max,ECF_, *t*_max,ECF_, *C*_max,B1_, and *t*_max,B1_. We see that *C*_max_,_ECF_ and *t*_max_,_ECF_ are only affected by binding kinetics when *B*_1_^max^ is high (Tables 8 and 9). Then, *C*_max_,_ECF_ is smaller than the reference value. The extent of this decrease depends on the values of *k*_1on_ and *k*_1off_: with increasing *k*_1on_, *C*_max_,_ECF_ becomes lower, while with increasing *k*_1off_, *C*_max_,_ECF_ becomes higher. Likewise, *t*_max_,_ECF_ generally increases with high *B*_1_^max^. It slightly decreases with higher *k*_1on_ when $\frac{k_{1 off}}{k_{10n}}\ge 100$_._

**Table 8:**
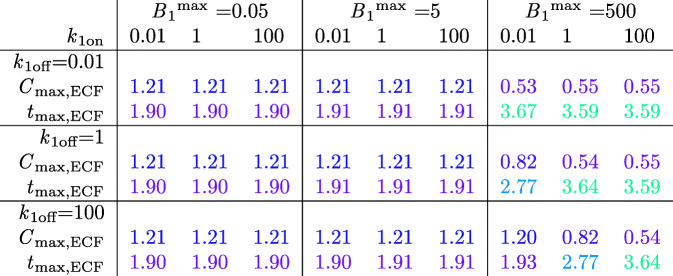
Impact of Brain Binding Site Concentrations on Brain ECF PK of Unbound Drug

The effects of *B*_1_^max^ (given in 10^−2^ μmol L^−1^), *k*_1on_ (given in (μmol L^−1^)s^−1^) and *k*_1off_ (given in 10^−2^ s^−1^) on *C*_ECF_ (given in μmol L^−1^) are shown. *C*_max,ECF_ and *t*_max_,_ECF_ (given in 10^4^ s) are shown. Colours are added to increase the readability of the table. Red indicates the lowest values of *C*_max_,_ECF_ and *t*_max_,_ECF_ and green indicates the highest values of *C*_max_,_ECF_ and *t*_max_,_ECF_. The values in between are coloured according to a 20-shades red-to-green colour bar based on the log values of the data

**Table 9:**
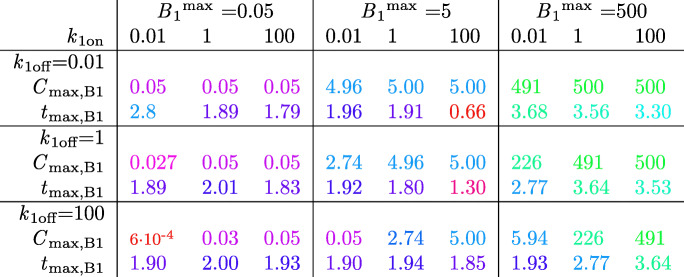
Impact of Brain Binding Site Concentrations on Brain ECF PK of Drug Bound to Specific Binding Sites

Effect of *B*_1_^max^ (given in 10^−2^ μmol L^−1^), *k*_1on_ (given in μmol L^−1^ s^−1^) and *k*_1off_ (given in 10^−2^ s^−1^) on *B*_1_ (given in μmol L^−1^). *C*_max_,_B1_ and *t*_max_,_B1_ (given in 10^4^ s) are shown. Colours are added to increase the readability of the table. Red indicates the lowest value and green indicates the highest value. Red indicates the lowest values of *C*_max_,_B1_ and *t*_max,B1_ and green indicates the highest values of *C*_max,B1_ and *t*_max,B1_. The values in between are coloured according to a 20-shades red-to-green colour bar based on the log values of the data

Obviously, *C*_max_,_B1_ is larger for higher values of *B*_1_^max^ (Table 9). Additionally, with a ratio of $\frac{k_{1 off}}{k_{10n}}\ge 100$, *C*_max_,_B1_ is smaller than *B*_1_^max^. The value of *t*_max_,_B1_ decreases with higher *k*_1on_ when *k*_1off_ is low. It increases with higher *k*_1on_ when *k*_1off_ and *B*_1_^max^ are high (lower right corner). In most other cases (except for when *B*_1_^max^ and *k*_1off_ are set to their reference values), *t*_max_,_B1_ first increases but then decreases with higher *k*_1on_. In all cases, except for when *k*_1on_ = 0.01 μmol L^−1^ s^−1^ and *k*_1off_ = 100·10^−2^ s^−1^, *t*_max,B1_ greatly increases when *B*_1_^max^ = 500·10^−2^ μmol L^−1^.

Next, as spatial differences in binding site concentrations may also occur on a small scale, we study the effect of local differences in binding site concentration *within* the ‘reference’ 3D brain unit network, with parameter values corresponding to the reference values given in Table 3, on the distribution of a drug within the network. We only assign specific binding sites to the 2x2x2 left, front and bottom units and thus set B_1max_ = 0 for x > 2d_cap_ + 4r, y > 2d_cap_ + 4r and z > 2d_cap_ + 4r. In addition, we study how different values of *B*_1_^max^ and *k*_1on_ in the units containing binding sites affect local distribution within the entire 3D brain unit network. Figures 7 and 8 show the spatial distribution profiles of *C*_ECF_ and *B*_1_, respectively. There, *C*_ECF_ is substantially smaller in the units with binding sites when either *B*_1_^max^ or *k*_1on_ is high (Fig. 7). In addition, *B*_1_ increases in the areas close to the capillaries relative to the areas in the middle of the units, furthest from the capillaries for large values of *B*_1_^max^ or *k*_1on_ (Fig. 8). When both *B*_1_^max^ and *k*_1on_ are set at their reference values, *B*_1_ is distributed equally over space.

To conclude, changes in the kinetics of drug binding to specific binding sites most impact free and bound drug concentrations when *B*_1_^max^ is high. These results imply that for drugs targeting the cannabinoid type 1 (CB1) receptor or the dopamine D1 receptor, *C*_max_,_ECF_ is lower but *t*_max,ECF_ is higher in the striatum, relative to other sites of the brain, because CB1 receptor concentration is highest in the striatum. This is particularly the case for drugs that strongly associate with the cannabinoid receptor (drugs that have a high value of *k*_1on_ and a low value of *k*_1off_).

### Combining Properties

In this section we study the effects of BBB transport (section 3.2) and drug binding kinetics (section 3.3), combined with other drug distribution processes, including brain capillary blood flow, diffusion and brain ECF bulk flow, on brain ECF PK. To this purpose, we show the impact of combinations of parameter changes on brain ECF PK.

Figure 9 shows values of *C*_max,ECF_ in the presence of combinations of low and high values of v_blood_, *P*, *T*_m-in_, *T*_m-out_, D*, v_ECF_ and in the absence or presence of binding. We now summarize the results given in Fig. 9. A change from high *P* to low *P* generally corresponds to a decrease in *C*_max,ECF_. The presence of active efflux (*T*_m − out_ > 0) enlarges this decrease, while a low value of D* or a lack of binding sites reduces this decrease. In addition, as discussed in section 3.2, in the presence of active influx a decrease in *P increases C*_max,ECF_, which is opposite to the general finding of this study.

Active influx induces an increase in *C*_max,ECF_, which is further affected by a low value of v_blood_ (lower increase), a high value of P (slightly higher or much lower increase, depending on the value of *T*_m-in_), the presence of active efflux (slightly lower or much lower increase, depending on the value of *T*_m-out_), a low value of D* (slightly higher increase) and the absence of binding sites (slightly higher increase). On the contrary, active efflux induces a decrease in *C*_max,ECF_, which is further affected by a low value of *P* (larger decrease) and the presence of active influx (smaller decrease or increase, depending on the value of *T*_m-in_). A reduction in D* with respect to the reference value corresponds to a slight increase in *C*_max,ECF_. Thereby, it counteracts the effects of decreases in *P* and *T*_m-in_ and an increase in *T*_m-out_, which all lower *C*_max,ECF_. In contrast, a decrease in v_ECF_, does not impact *C*_max,ECF_. Finally, the absence of binding sites, in general, slightly increases *C*_max,ECF_.

We have also assessed the effects of combinations of parameters on *t*_max,ECF_, *C*_max,B1_, *t*_max_,_B1_, of which the data are summarized in Appendix I. In short, a low value of *P* corresponds to a high value of *t*_max,ECF_, while high values of *P* and/or *T*_m-out_ correspond to a low value of *t*_max,ECF_. (Appendix I, Fig. 1). Both a decrease in D* and the absence of binding sites also lower *t*_max,ECF_.

Then, values of *C*_max,B1_ are mostly unaffected by parameter changes, with the exception of no binding (*C*_max,B1_ = 0), a low value of *P* (a slightly lower *C*_max,B1_) and a high value of *T*_m-out_ (a slightly lower *B*_max,1_), see Appendix I, Fig. 2. Finally, the parameter combinations affect values of *t*_max,B1_ similarly as they affect values of *t*_max,ECF_, see Fig. 3 in Appendix I.

We conclude that changes in BBB transport including BBB permeability, BBB active influx and BBB active efflux affect brain ECF PK most. Additionally, decreases in brain ECF diffusion, which is likely impaired due to leakage of blood-derived cells into the brain ECF as occurs in many brain diseases (7), slightly affect brain ECF PK by increasing *C*_max,ECF_.

### Examples for a Number of Existing Drugs

We next study how brain ECF PK of 3 existing drugs with distinctive physicochemical properties (morphine, phenytoin and methrotrexate) is affected by changes in parameters that may be related to brain disease. Morphine is a drug with a relatively low BBB permeability that is subject to both active efflux and active influx across the BBB (25). Phenytoin is a drug that easily crosses the BBB via passive transport and is not subject to significant active transport and has high non-specific binding (26,27). Finally, methotrexate is a drug with a very low BBB permeability that is subject to active efflux (28). The drug-specific parameter values for morphine, phenytoin and methotrexate are summarized in Table 10, while all other parameters are given in Table 3. The values of *B*_2max_, *k*_2on_ and *k*_2off_ (Table 10) are, due to a lack of experimental data on non-specific binding kinetics, based on the brain ‘fraction unbound’$\left(\frac{\mathrm{Free}\ \mathrm{drug}\ \mathrm{in}\ \mathrm{brain}}{\mathrm{Total}\ \mathrm{drug}\ \mathrm{in}\ \mathrm{brain}}\right)$ reported in literature (29,30): the values of *B*_2max_, *k*_2on_ and *k*_2off_ have been tuned until, in the presence of a constant value of *C*_pl_, the 3D brain unit model showed a value of the fraction unbound (calculated as C_ECF_ /(C_ECF_+B_1_ + B_2_) that was identical to the value reported in literature. Figure 10 shows morphine, phenytoin and methotrexate brain ECF PK under reference conditions with all drug-specific parameter values as in Table 10 (Fig. 10, black lines) and with parameters that reflect changes in BBB transport (Fig. 10, left) or binding site concentrations (Fig. 10, right). To investigate the relation between drug within the blood plasma (measurable) and within the brain ECF (often not measurable), blood plasma PK (calculated with parameters as in Table 3) is taken the same for all three drugs. We observe from Fig. 10 (left) that morphine brain ECF PK is highly affected by several changes in BBB transport. An increase in BBB permeability (high P) only slightly increases *C*_max,ECF_, which reflects the fact that morphine brain ECF PK is mostly regulated by BBB active influx and active efflux. Inhibition of influx (*T*_m − in_ = 0) leads to a lower *C*_max,ECF_ and a faster decrease of *C*_max,ECF_. In contrast, inhibition of efflux increases *C*_max,ECF_, but does not change the shape of the brain ECF concentration-time profile of morphine. An increase in efflux lowers *C*_max,ECF_, but, again, does not change the shape of the brain ECF PK of morphine. Inhibition of both influx and efflux results in a higher *C*_max,ECF_, but with a concentration-time profile that is similar in shape to the concentration- time profile when only influx is inhibited. While morphine brain ECF PK is greatly affected by changes in BBB transport, it is unaffected by changes in concentrations of both specific and non-specific binding sites (Fig. 10, right). In contrast to morphine concentrations, phenytoin concentrations are hardly affected by increases in *P*, as, by default, phenytoin easily crosses the BBB (Fig. 10). In addition, while phenytoin brain ECF PK is unaffected by decreases in concentrations of both specific and non-specific binding sites, phenytoin brain ECF PK is affected by an *increase* in *B*_2_^max^ (Fig. 10): an increase in *B*_2_^max^ slightly decreases *C*_max,ECF_, while it increases *t*_max,ECF_ (Fig. 10).

**Table 10:** Properties of three existing drugs targeting the brain

	Morphine	Phenytoin	Methotrexate
*P* (·10^−7^ m s^−1^)	0.42	13	0.001
*T*_m-in_ (·10^−7^ μmol s^−1^)	0.384	0	0
*K*_m-in_ (·10^2^ μmol L^−1^)	0.000348	0	0
*T*_m-out_ (·10^−7^ μmol s^−1^)	14	33	2.1
*k*_m-out_ (·10^2^ μmol L^−1^)	0.15	30	1
*B*_1_^max^ (·10^−2^ μmol L^−1^)	0.05	0.05	0.005
*k*_1on_ ((μmol L^−1^)s^−1^)	0.014	0.025	37
*k*_1off_ (·10^−2^ s^−1^)	0.23	50	0.033
*B*_2_^max^ (·10^1^ μmol L^−1^)	0.25	0.05	0.05
*k*_2on_ (·10^−2^(μmol L^−1^)s^−1^)	1	30	1
*k*_2off_ (s^−1^)	1	1	1

All values are relative to the reference values in Table 3. The influx parameters for morphine are taken from (25), the efflux parameters for morphine and methotrexate are based on (25,28). Data on permeability originate from (29). Data on non-specific binding is based on data of free drug fraction in (29,30). Data on specific binding kinetics for all drugs originate from (31)

Finally, methotrexate concentrations within the brain ECF are very low due to its low BBB permeability and high efflux. Therefore, both an increase in *P* (Fig. 10, down left, green line) and an inhibition of efflux (Fig. 10, down left, red line) lead to a higher value of *C*_max,ECF_. On the other hand, a high value of *T*_m-out_ results in a lower value of *C*_max,ECF_. Increases in concentrations of specific and, particularly, non-specific binding sites correspond to great increases in *t*_max,ECF_ and only slight decreases in *C*_max,ECF_ (Fig. 10, down right). In similar fashion, the absence of both specific and non-specific binding sites decreases *t*_max,ECF_, but only slightly increases *C*_max,ECF_ (Fig. 10, down right). In conclusion, our simulations predict that morphine PK is greatly affected by changes in BBB active influx and active efflux and thus, morphine PK likely changes in diseases like Alzheimer’s, ALS, epilepsy and brain cancer. Finally, for methotrexate the model predicts that an increase in BBB permeability or a disruption of BBB active efflux, like may occur in stroke, increases *C*_ECF_, while an increase in BBB active efflux, like may occur in ALS, epilepsy and brain cancer, decreases *C*_ECF_. Both phenytoin and methotrexate are affected by high concentrations of non-specific binding sites, which may differ within the brain.

## Discussion

We have developed a mathematical model that describes the spatial distribution of a drug within a 3D brain unit network. The 3D brain unit network model is an extension of our earlier 3D brain unit model (submitted to PLOS Computational Biology). It enables the study of spatial concentration differences at two levels:

1) The *entire* 3D brain unit network in health and disease conditions. Disease conditions are reected by differences in parameters that may arise due to differences in brain capillary density (section 3.1), BBB transport (for example due to local disease, section 3.2) or local specific binding site density (section 3.3).

2) Local differences in parameters between units *within* the network, see Figs 8 and 9.

**Fig. 5:**
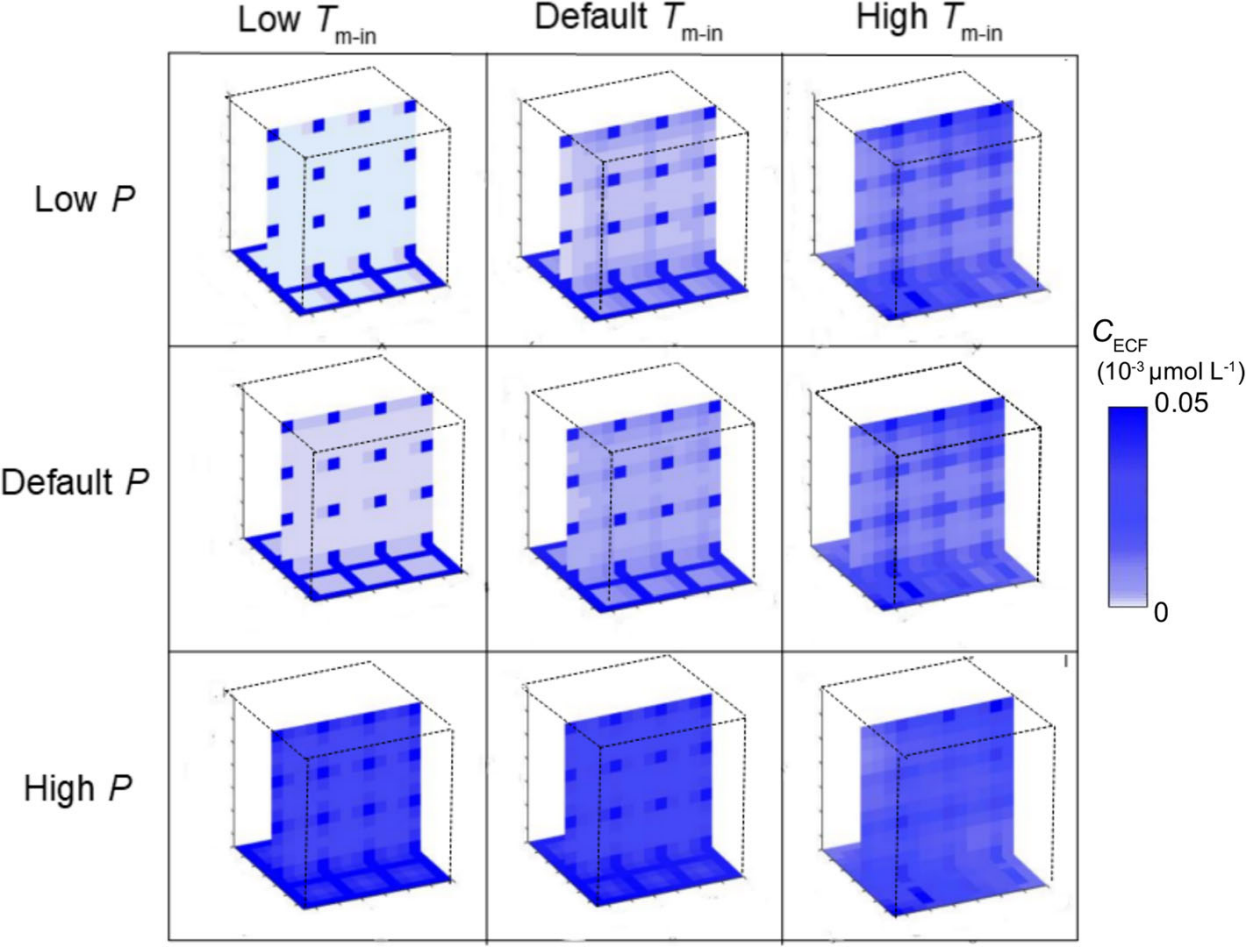
The effect of changes in passive BBB permeability and active BBB influx on unbound drug concentrations within the brain ECF. The BBB permeability, *P* is set at low (0.01·10^−7^ m s^−1^), at its reference value (0.1·10^−7^ m s^−1^) or high (1·10^−7^ m s^−1^). The active BBB influx transporter velocity, *T*_m-in_ is set at 0.1·10^−7^ μmol s^−1^ (low), 1·10^−7^ μmol s^−1^ (reference value) or 10·10^−7^ μmol s^−1^ (high). Darker shades of blue correspond to higher concentrations of unbound drug within the brain ECF. Distribution profiles are shown at *t* = 50 s.

In our studies we have focused on the effect of brain capillary density, BBB transport and drug binding kinetics on brain ECF PK. First, in section 3.1, we have studied the effect of brain capillary density on brain ECF PK. The brain capillary density is often related to other properties, like the spatial organization of the blood vessels, changes in brain capillary diameter, or local obstructions. For simplicity, we have chosen to base the brain capillary density only on the distance between the capillaries, *d*_cap_. We have found a positive correlation between brain capillary density and drug concentrations within the brain ECF, for low values of BBB permeability (Fig. 5). No significant affect of brain capillary density was observed for *high* values of BBB permeability. The relationship between capillary density and drug uptake was investigated in an experimental study on drug distribution within the murine brain (32). There, a positive correlation between capillary density and drug uptake was found within the brain of mice lacking the active transporter P-glycoprotein for three drugs with different values of BBB permeability. Unlike in our study, brain capillary density *did* affect drug uptake into the brain ECF with higher values of BBB permeability. However, the study was performed with the brain perfusion technique and focused on initial drug uptake into the brain, while in our model we also take the processes after drug uptake into account, i.e. drug distribution within and elimination from the brain. It is likely that in the presence of a high permeability, diffusion contributes to a quick equilibration of drug within the blood plasma and the brain ECF, but this requires further investigation.

**Fig. 6:**
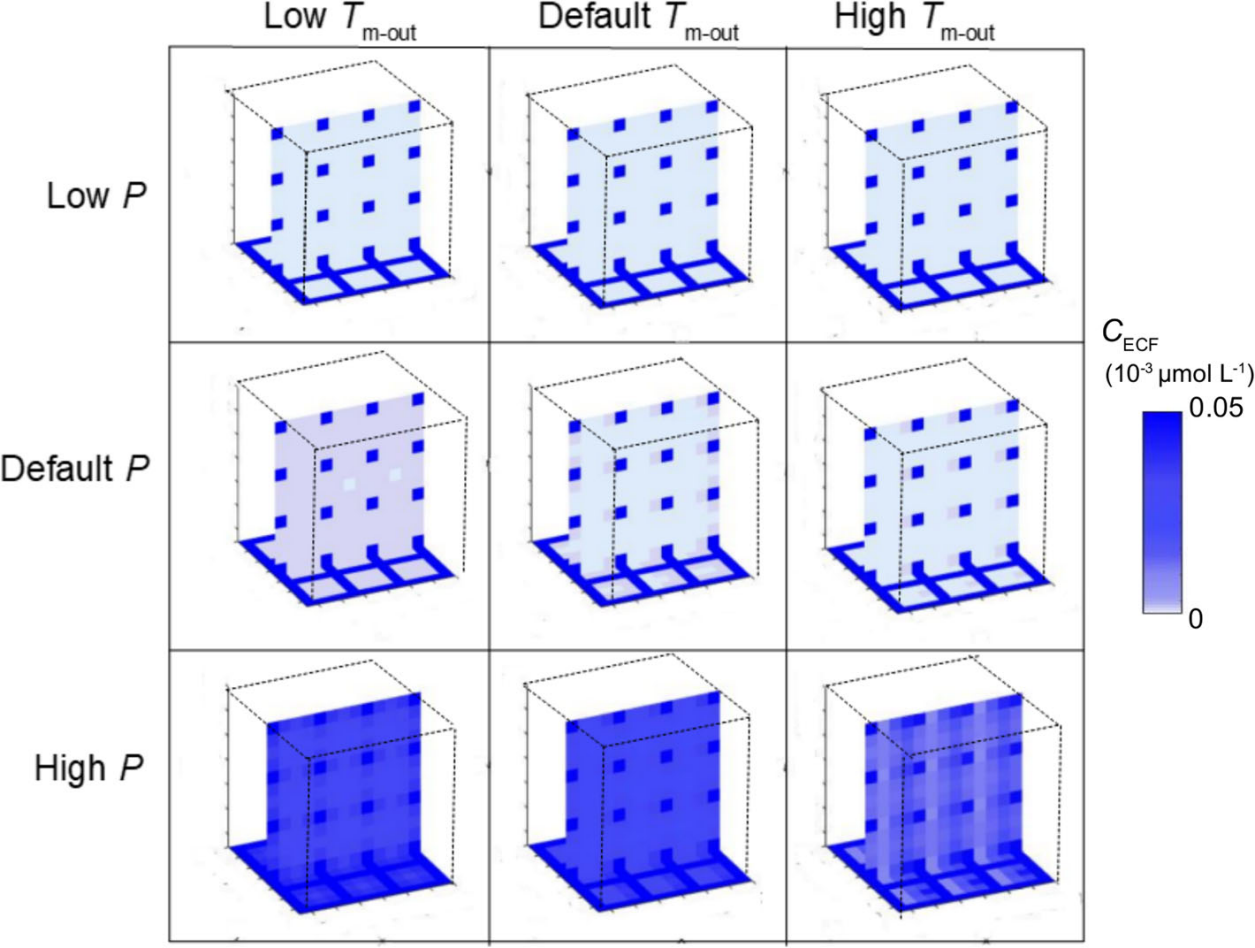
The effect of changes in passive BBB permeability and active efflux on unbound drug concentrations within the brain ECF. The BBB permeability, *P* is set at low (0.01·10^−7^ m s^−1^), at its reference value (1·10^−7^ m s^−1^) or high (100·10^−7^ m s^−1^). The active BBB efflux transporter velocity, *T*_m-out_ is set at 0.1·10^−7^ μmol s^−1^ (low), 1·10^−7^ μmol s^−1^ (reference value) or 10·10^−7^ μmol s^−1^ (high). Higher intensities of blue correspond to higher concentrations of unbound drug within the brain ECF. Distribution profiles are shown at t = 50 s

**Fig. 7:**
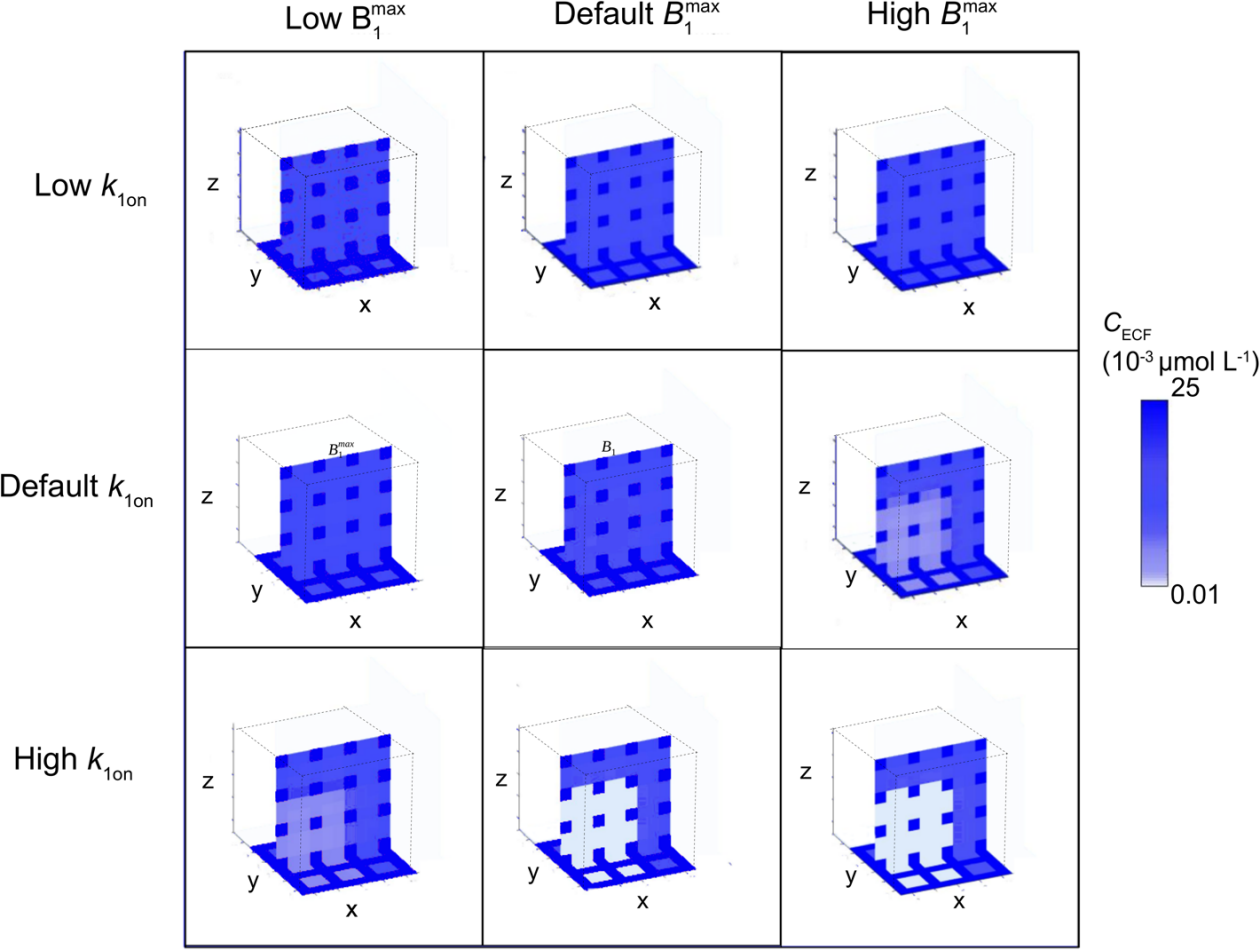
The effect of changes in specific binding site density and association rate constant on unbound drug concentrations within the brain ECF. The target concentration, *B*_1_^max^ is set at 0.01·10^−2^ μmol L^−1^ (low), 1·10^−2^ μmol L^−1^ (reference value) or 100·10^−2^ μmol L^−1^. The association rate constant of drug with its target, *k*_1on_ is set at 0.01 μmol L^−1^ s^−1^ (low), 1 μmol L^−1^ s^−1^ (reference value) or 100 μmol L^−1^ s^−1^ (high). Higher intensities of blue correspond to higher concentrations of unbound drug within the brain ECF. Distribution profiles are shown at t = 100

**Fig. 8:**
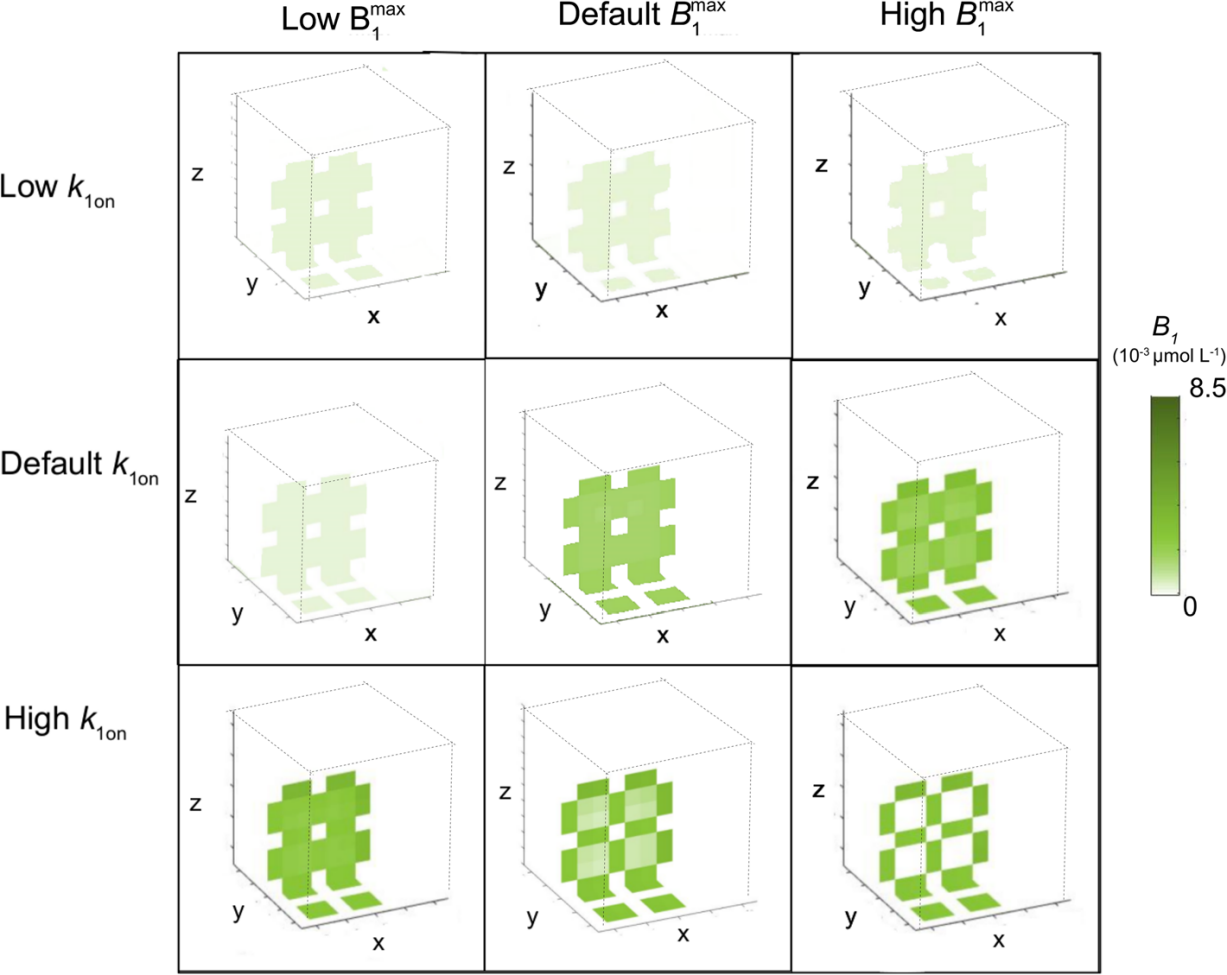
The effect of changes in specific binding site density and association rate constant on concentrations of target-bound drug within the brain ECF. The total target concentration, *B*_1_^max^ is set at 0.01·10^−2^ μmol L^−1^ (low), 1·10^−2^ μmol L^−1^ (reference value) or 100·10^−2^ μmol L^−1^ (high). The association rate constant of drug with its target, *k*_1on_ is set at 0.01 μmol L^−1^ s^−1^ (low), 1 μmol L^−1^ s^−1^ (reference value) or 100 μmol L^−1^ s^−1^ (high). Higher intensities of green correspond to higher concentrations of drug bounds to targets facing the brain ECF. White corresponds to a concentration of bound drug that equals zero, like in the blood plasma of the brain capillaries, or, in case of strong binding to a high concentration of specific binding sites (bottom right) in the middle of the units. Distribution profiles are shown at t = 100

**Fig. 9:**
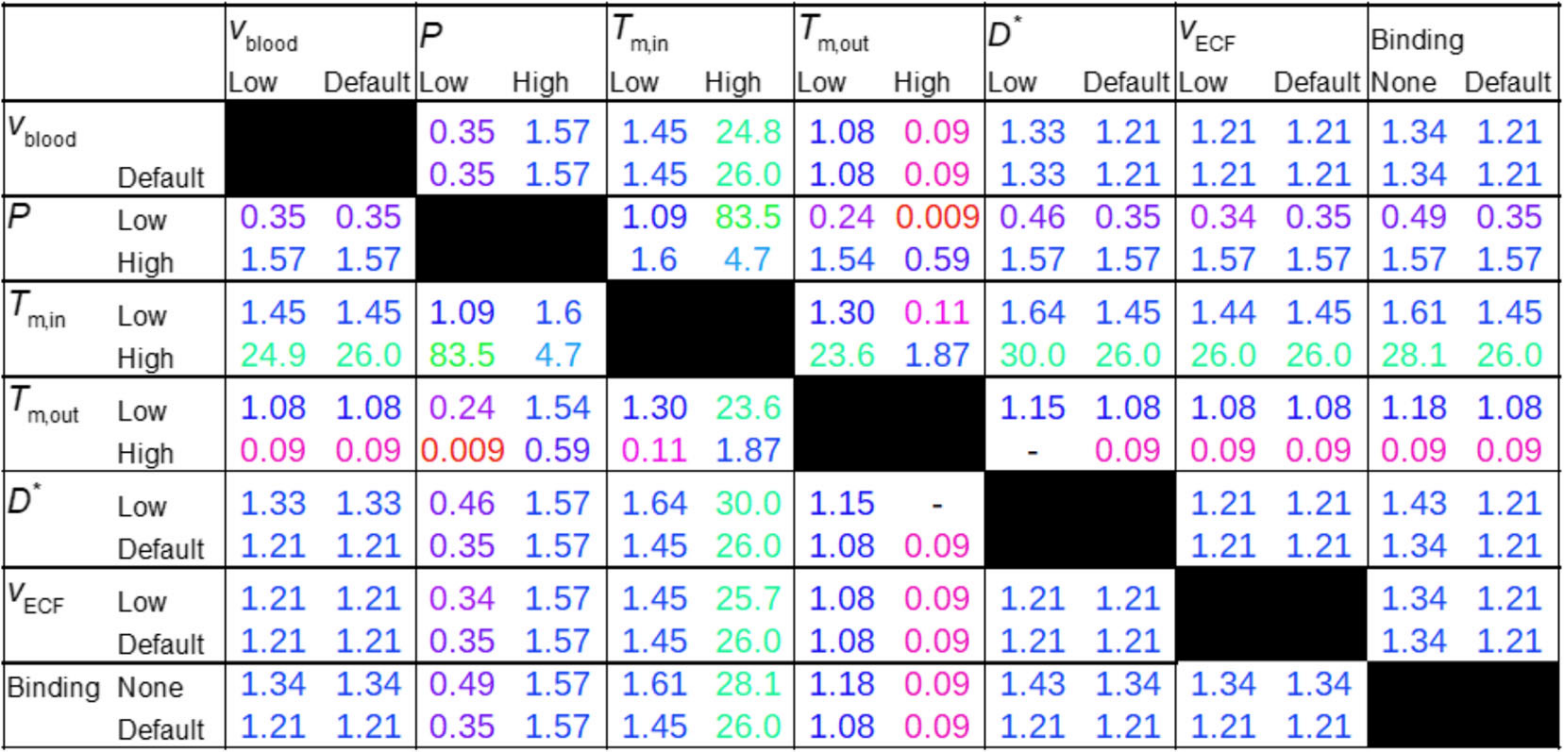
Integration of properties. The impact of combinations of parameters on *C*_max,ECF_ is shown. Reference parameter values are as in Table 3. Low v_blood_ = 0.5·10^−4^ m s^−1^, low *P* = 0.01·10^−7^ m s^−1^, high *P* = 1·10^−7^ m s^−1^, low *T*_m-in_ = 0.1·10^−7^ μmol L^−1^ s^−1^, high *T*_m-in_ = 10·10^−7^ μmol L^−1^ s^−1^, low *T*_m-out_ = 0.1·10^−7^ μmol L^−1^ s^−1^, high *T*_m-out_ = 10·10^−7^ μmol L^−1^ s^−1^, low D* = 0.05·10^−10^ m^2^ s^−1^, low v_ECF_ = 0.05·10^−10^ m s^−1^. Binding includes the concentrations of both specific and non-specific binding sites, i.e. when binding is none, *B*_1_^max^ = 0 and *B*_2_^max^ = 0. For clarity, the table is symmetric, such that both the effect of parameter A on parameter B and the effect of parameter B on parameter A can be easily assessed. Colours are added to increase the readability of the table. Red indicates the lowest values of *C*_max,ECF_ and *t*_max,ECF_ and green indicates the highest values of *C*_max,ECF_ and *t*_max,ECF_. The values in between are coloured according to a 20-shades red-to-green colour bar based on the log values of the data.

**Fig. 10:**
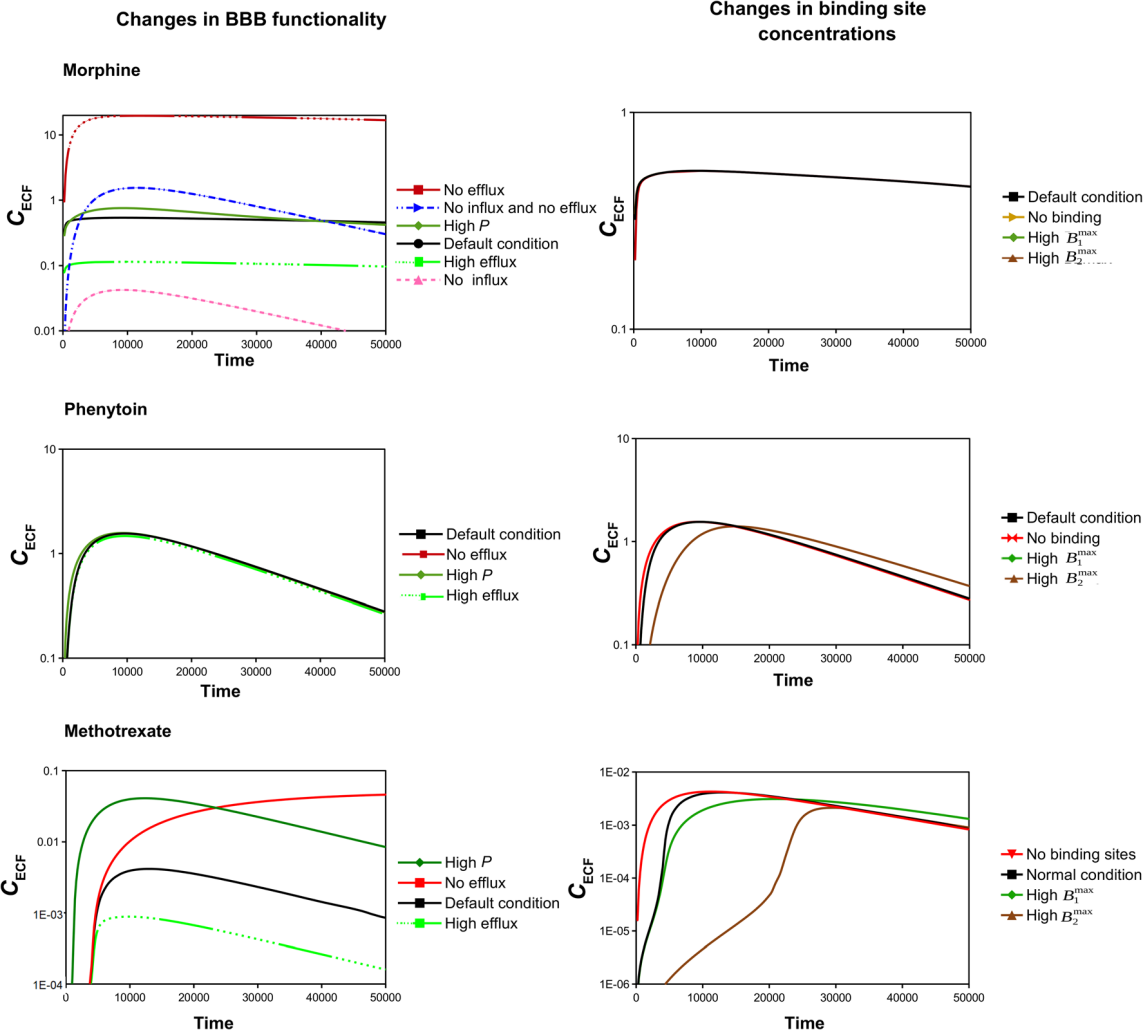
Brain ECF PK of existing drugs under health and disease conditions. Brain ECF PK of morphine (top), phenytoin (middle) and methotrexate (low) is shown under reference conditions (in black) and under conditions of changes in BBB permeability and binding properties (in green and red). Left: Effect of changes in BBB transport on brain ECF PK. The BBB permeability is increased (green), or decreased (red). For compounds with active influx and/or efflux, the BBB permeability is also increased (green) or decreased (green). Right: Effect of changes in binding site concentrations on brain ECF PK. Either the concentration of specific or non-specific binding sites is zero (no binding, red) or high (green for *B*_1_^max^, brown for *B*_2_^max^).

Changes in parameters related to BBB transport, as may occur in disease conditions, affect brain ECF PK, including *C*_max,ECF_, *t*_max,ECF_, and the spatial distribution of a drug, within the 3D brain unit network (Section 3.2). There, BBB active transport depends on the permeability of the BBB to the drug and the impact of both active influx and active efflux decreases with a higher BBB permeability. Indeed, mostly drugs that have difficulties crossing the BBB (due to high polarity and high molecular weight) are shown to be significantly impacted by active efflux (33).

In section 3.3 we have shown that specific binding site density affects brain ECF PK of unbound drug and drug bound to specific binding sites within the 3D brain unit network. Moreover, we have shown how local differences in specific binding site concentration affect the distribution of *C*_ECF_ within the 3D brain unit network. The distribution profiles of *C*_ECF_ and *B*_1_ are particularly affected by *B*_1_^max^, as is shown in Tables 7 and 8. In addition, increasing *k*_1on_ has similar effects on *C*_ECF_ and *B*_1_ as decreasing *k*_1off_. This is in line with recent studies stating that target association and dissociation are equally important (34,35).

Finally, in section 3.4, we have shown how a combination of properties (for example, the combination of an increased BBB permeability and a decreased diffusion, as occurs in many brain diseases (7)) impacts *C*_ECF_. We situated how different BBB and brain distribution parameter values (due to local disease and location) a_ect the concentration-time profiles of 3 existing drugs. We find that morphine brain ECF PK is mainly determined by the balance between active influx and active efflux, as has been shown before (25). Therefore, the shape of the concentration-time profile greatly changes when BBB influx or efflux is affected, but not when BBB permeability is increased (Fig. 10). Phenytoin brain ECF PK within the 3D brain unit network is hardly affected by BBB transport. This is partly in line with experimental findings that epileptic-seizure-induced increases in BBB transport do not increase, but, interestingly, rather decrease unbound phenytoin concentrations in rat brain ECF (36). This decrease is possibly caused by enhanced extracellular protein binding related to seizure induction (36,37).

Methotrexate concentrations are affected by both changes in BBB transport and high concentrations of binding sites (Fig. 10). In addition, experiments have shown that methrotexate concentrations are affected by intra-extracellular exchange: upon entering cells, methotrexate is converted into polyglutamate methotrexate by metabolic enzymes (38). This leads to `trapping’ of methotrexate in the cells, thereby greatly affecting the concentrations of methotrexate in the brain ECF. In our model, however, we do not distinguish between intracellular and extracellular compartments and therefore we have not taken intracellular trapping of methotrexate into account. Our future goal is to distinguish between intracellular and extracellular compartments and binding sites.

The focus of our model is on drug distribution within the brain, after transport of drug into the brain from the brain vasculature. Therefore, the 3D brain unit network model represents a small region of interest, where the brain capillaries, which are the major site of exchange between the blood and the brain, surround the brain ECF. In the future, one or multiple 3D brain unit networks can be implemented in a large-scale 3D model of the brain, that describes drug transport into and within larger areas of the brain. Due to the large scale of such a model, it is feasible that 3D brain unit networks only describe a small region of interest, which is generally the area the drug is targeting, like the area of local disease or the area where most drug targets are located. The other areas should then be described in less detail, i.e. by larger units describing regions where differences are non-existent or negligible.

We have shown that our model is suitable for the study of drug distribution within a small part of the brain. The parameters inherent to this specific area of interest can be easily put into our model to study drug distribution within this area. In addition, data on particular existing drugs can be implemented by using parameters inherent to this drug (see Table 10). As such, the 3D brain unit network model enables the study of the distribution of specific drugs within a specific area of interest in the brain. In addition, it enables the study on how spatial distribution is affected by changes in parameters, as induced by differences in location or by local disease. In summary, the 3D brain unit network model provides an excellent starting point to study the distribution of a drug within the brain and assess the effect of spatial differences within the brain on spatial distribution of a drug within the brain.

## Electronic supplementary material

(DOCX 2.30 MB)

## Abbreviations

BBB: blood-brain barrier
brain ECF: brain extracellular fluid
PK: pharmacokinetics
