# Supplementary material for: The 3D Brain Unit Network Model to Study Spatial Brain Drug Exposure under Healthy and Pathological Conditions
Source: Pharm Res. 2020 Jul 9;37(7):137. doi: 10.1007/s11095-020-2760-y (PMC7347686; doi:10.1007/s11095-020-2760-y)
Supplement: Supplementary file 1 — (DOCX 2.30 MB) [file 11095_2020_2760_MOESM1_ESM.docx]

**Appendix I - Nondimensionalization of the model**


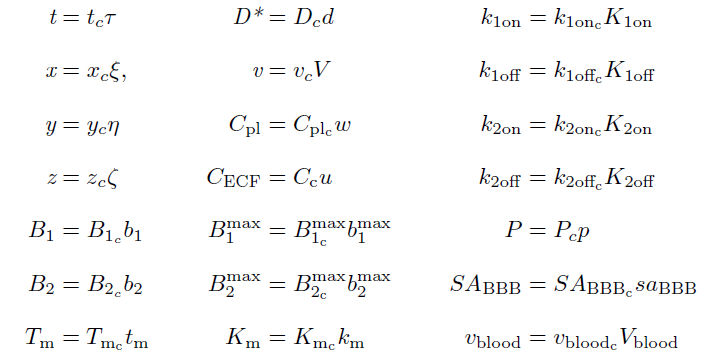

We can make equations (2-14) dimensionless by introducing a change of variables. Here, the original variables are scaled to dimensionless variables by scaling with a characteristic, dimensional scale. We set:

where


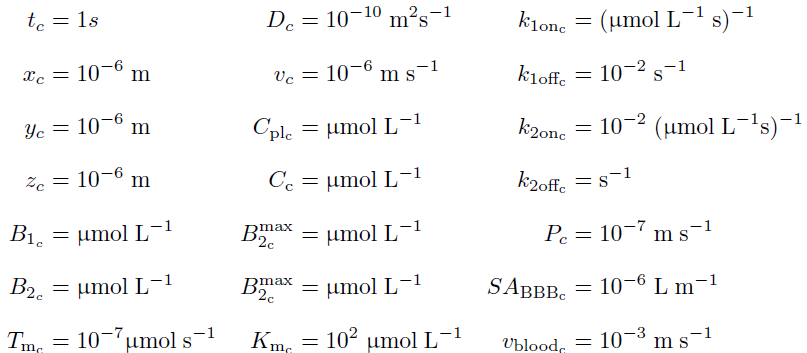


This leads to the following dimensionless equation for drug in the blood plasma (example based on equation (2), but similar for equations (3)-(4)):

and the following system of dimensionless equations for drug within the brain ECF (for equation (6)):


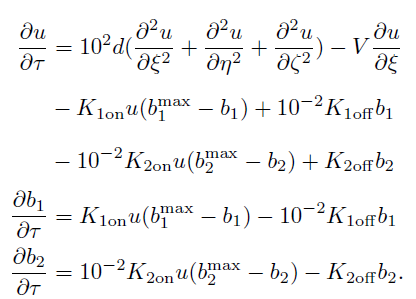


The corresponding boundary conditions (equations (10)-(11), example for equation (10), but similar for equations (11)) are given by:


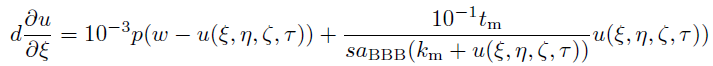


for ξ=0 and x=1.

The initial conditions become:


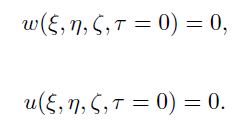


**Appendix II - Additional data**


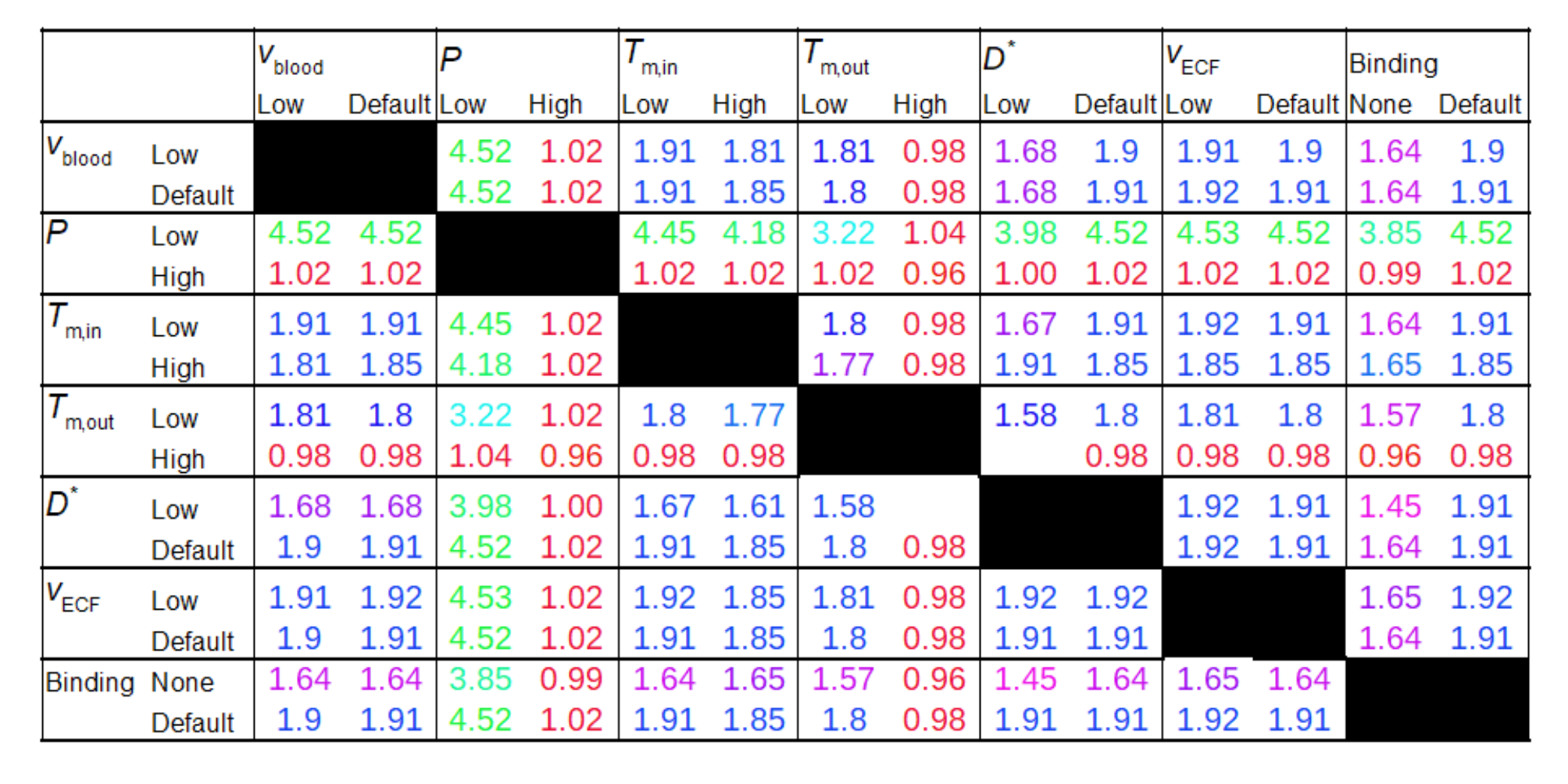


**Fig. 1 Integration of properties.** The impact of combination of parameters on *t*_max,ECF_ is shown. Reference parameter values are as in Table 3. Low *v*_blood=_ 0.5*·*10^-4^ m s^-1^, low *P* = 0.01*·*10^-7^ m s^-1^, high *P* = 1·10^-7^ m s^-1^, low *T*_m-in_ = 0.1·10^-7^ µmol L-1s^-1^, high *T*_m-in_ = 10·10^-7^ µmol L-1s^-1^, low *T*_m-out_ = 0.1·10^-7^ µmol L-1s^-1^, high *T*_m-out_ = 10·10^-7^ µmol L-1s^-1^, low *D^*^ =* 0.05·10^-10^ m^2^s^-1^, low *v*_ECF_ = 0.05·10^-10^ m s^-1^. Binding includes the concentrations of both specific and non-specific binding sites, i.e. when binding is none, B_1_^max^=0 and B_2_^max^=0. For clarity, the table is symmetric, such that both the effect of parameter A on parameter B and the effect of parameter B on parameter A can be easily assessed. Colours are added to increase the readability of the table. Red indicates the lowest value of *t*_max,ECF_ and green indicates the highest value of *t*_max,ECF_. The values in between are coloured according to a 20-shades red-to-green colour bar based on the log values of the data.


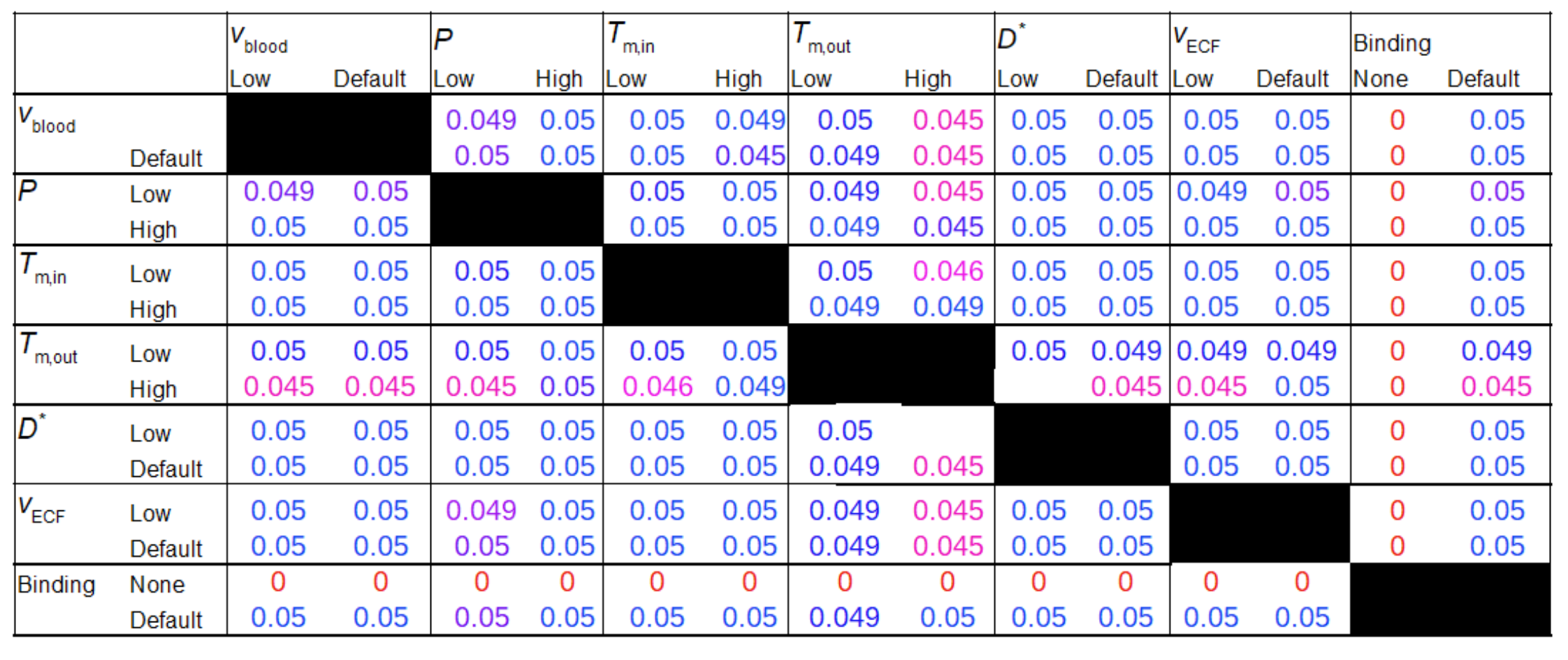


**Fig. 2 Integration of properties.** The impact of combination of parameters on *C*_max,B1_ is shown. Reference parameter values are as in Table 3. Low *v*_blood=_ 0.5*·*10^-4^ m s^-1^, low *P* = 0.01*·*10^-7^ m s^-1^, high *P* = 1·10^-7^ m s^-1^, low *T*_m-in_ = 0.1·10^-7^ µmol L-1s^-1^, high *T*_m-in_ = 10·10^-7^ µmol L-1s^-1^, low *T*_m-out_ = 0.1·10^-7^ µmol L-1s^-1^, high *T*_m-out_ = 10·10^-7^ µmol L-1s^-1^, low *D^*^ =* 0.05·10^-10^ m^2^s^-1^, low *v*_ECF_ = 0.05·10^-10^ m s^-1^. Binding includes the concentrations of both specific and non-specific binding sites, i.e. when binding is none, B_1_^max^=0 and B_2_^max^=0. For clarity, the table is symmetric, such that both the effect of parameter A on parameter B and the effect of parameter B on parameter A can be easily assessed. Colours are added to increase the readability of the table. Red indicates the lowest value of *C*_max,B1_ and green indicates the highest value *C*_max,B1_. The values in between are coloured
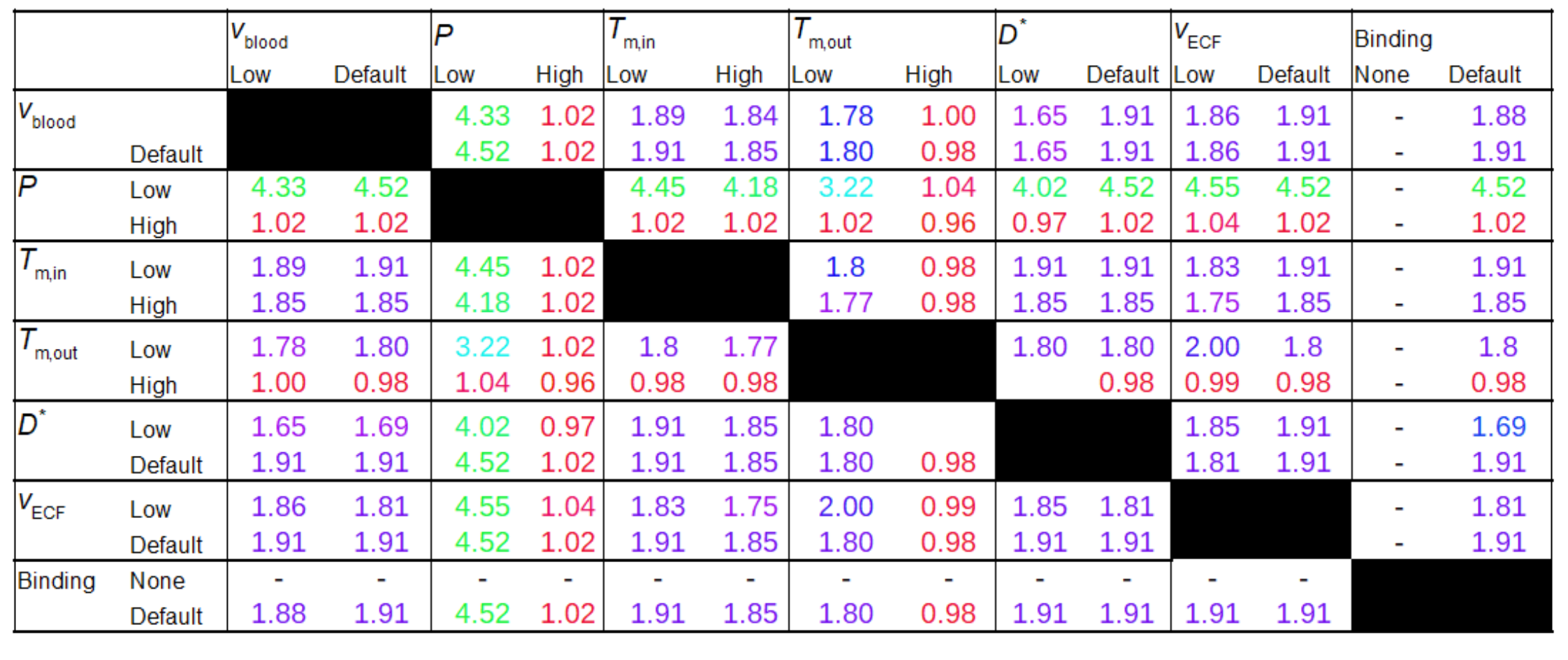
according to a 20-shades red-to-green colour bar based on the log values of the data.

**Fig. 3 Integration of properties.** The impact of combination of parameters on *t*_max,B1_ is shown. Reference parameter values are as in Table 3. Low *v*_blood=_ 0.5*·*10^-4^ m s^-1^, low *P* = 0.01*·*10^-7^ m s^-1^, high *P* = 1·10^-7^ m s^-1^, low *T*_m-in_ = 0.1·10^-7^ µmol L-1s^-1^, high *T*_m-in_ = 10·10^-7^ µmol L-1s^-1^, low *T*_m-out_ = 0.1·10^-7^ µmol L-1s^-1^, high *T*_m-out_ = 10·10^-7^ µmol L-1s^-1^, low *D^*^ =* 0.05·10^-10^ m^2^s^-1^, low *v*_ECF_ = 0.05·10^-10^ m s^-1^. Binding includes the concentrations of both specific and non-specific binding sites, i.e. when binding is none, B_1_^max^=0 and B_2_^max^=0. For clarity, the table is symmetric, such that both the effect of parameter A on parameter B and the effect of parameter B on parameter A can be easily assessed. Colours are added to increase the readability of the table. Red indicates the lowest value of *t*_max,B1_ and green indicates the highest value *t*_max,B1_. The values in between are coloured according to a 20-shades red-to-green colour bar based on the log values of the data.
